# Phytochemistry Meets Geochemistry—Blumenol C Sulfate: A New Megastigmane Sulfate from *Palicourea* *luxurians* (Rubiaceae: Palicoureeae)

**DOI:** 10.3390/molecules27217284

**Published:** 2022-10-26

**Authors:** Christoph Kornpointner, Nadine J. Hochenegger, Bao-Bao Shi, Andreas Berger, Johannes Theiner, Lothar Brecker, Johann Schinnerl

**Affiliations:** 1 Department of Organic Chemistry, University of Vienna, Währinger Strasse 38, A-1090 Vienna, Austria; 2 Institute of Chemical, Environmental and Bioscience Engineering, Technische Universität Wien, Getreidemarkt 9/166, A-1060 Vienna, Austria; 3 School of Pharmaceutical Sciences, South-Central MinZu University for Nationalities, Wuhan 430074, China; 4 Department of Botany and Biodiversity Research, University of Vienna, Rennweg 14, A-1030 Vienna, Austria; 5 Microanalysis Services, University of Vienna, Währinger Strasse 42, A-1090 Vienna, Austria

**Keywords:** Rubiaceae, *Palicourea luxurians*, Río Sucio, megastigmane, blumenol C sulfate, elemental analysis

## Abstract

There is a previously neglected influence of geochemical conditions on plant phytochemistry. In particular, high concentrations of dissolved salts can affect their biosynthesis of natural products. Detoxification is most likely an important aspect for the plant, but additional natural products can also give it an expanded range of bioactivities. During the phytochemical analysis a *Palicourea luxurians* plant collected in a sulfate-rich environment (near the Río Sucio, Costa Rica) showed an interesting natural product in this regard. The structure of this compound was determined using spectroscopic and computational methods (NMR, MS, UV, IR, CD, optical rotation, quantum chemical calculations) and resulted in a megastigmane sulfate ester possessing a β-ionone core structure, namely blumenol C sulfate (**1**, C_13_H_22_O_5_S). The levels of sulfur and sulfate ions in the leaves of the plant were determined using elemental analysis and compared to the corresponding levels in comparable plant leaves from a less sulfate-rich environments. The analyses show the leaves from which we isolated blumenol C sulfate (**1**) to contain 35% more sulfur and 80% more sulfate than the other samples. Antimicrobial and antioxidant activities of compound **1** were tested against *Escherichia coli*, *E. coli* ampR and *Bacillus subtilis* as well as measured using complementary in vitro FRAP and ATBS assays, respectively. These bioactivities are comparable to those determined for structurally related megastigmanes. The sulfur and sulfate content of the plant leaves from the sulfate-rich environment was significantly higher than that of the other plants. Against this background of salt stress, we discuss a possible biosynthesis of blumenol C sulfate (**1**). Furthermore, there appears to be no benefit for the plant in terms of extended bioactivities. Hence, the formation of blumenol C sulfate (**1**) probably primarily serves the plant detoxification process.

## 1. Introduction

Megastigmanes are oxygenated isonorterpenoids possessing a C_13_ carbon skeleton and are commonly known as oxidative degradation products from β-carotenoids [1]. Until the early 2010s approximately 150 different known megastigmanes have been reported [2]. This number is likely to have risen since then, although no further compilations of known compounds of this substance class have been published.

Apart from their unsubstituted forms, megastigmanes regularly occur glycosylated [3,4], carboxylated [5] or as epoxides [6]. Such derivatives are found in many plant species throughout the angiosperms [7]. Within the coffee family (Rubiaceae) megastigmanes are known from the rubiaceous species *Lasianthus fordii* Hance [8], *Guettarda speciosa* L. [9] and *Diplospora dubia* (Lindl.) Masam. [10,11], published under *Tricalysia dubia* (Lindl.) Ohwi. Megastigmane glycosides, bearing a sulfate group directly linked to the carbohydrate moiety, have been reported from *Ruellia patula* Jacq. (Acanthaceae) [12] and from *Garcinia mangostana* L. (Clusiaceae) [13]. In general, plant-derived compounds linked with a sulfate group occur rather seldom and most reports pertain to terpenoid and flavonoid glycosides with sulfate attached to the sugar moiety [11,14,15]. To best of our knowledge, naturally occurring megastigmane aglycones, which are directly linked to a sulfate group have not yet been reported.

Several megastigmanes and their derivatives are described to exhibit various bioactivities. For instance, they are known for their antimicrobial [16], antioxidant [4,17], antitumor [18] as well as antiviral activity [19]. Thus, they are of interest in phytochemical investigations, which also relate to the bioactivities of natural compounds.

**Figure 1:**
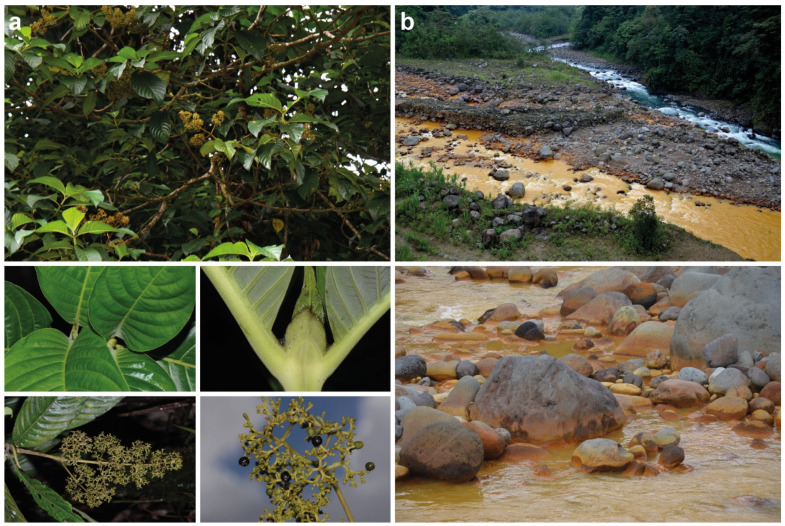
(**a**) Habitus, leaves, stipules and infructescence with fruits of *Palicourea luxurians* (Rubiaceae: Palicoureeae) growing on the steep riverbanks of Río Sucio; (**b**) Confluence of Río Sucio (brownish-yellowish, foreground) and Río Honduras (blue, background) near the bridge of Route 32 through pristine Braulio Carrillo National Park. The color of the river indicates deposits of schwertmannite, a yellowish-brownish sulfate containing iron oxide [27]. All photos: A. Berger.

In the framework of our phytochemical research on species of *Palicourea* (Rubiaceae: Palicoureeae; [20,21,22,23,24]) and related species, we recently studied an accession of *Palicourea luxurians* (Rusby) Borhidi (Figure 1a) collected near the confluence of the two rivers Río Sucio and Río Honduras in Costa Rica (Figure 1b; [24]). This species accumulates iridoids, and differs more notably from related congeners by accumulating alstrostine-type instead of strictosidine-type alkaloids otherwise characterizing the genus *Palicourea* [25,26].

In continuation of our previous studies, we now report an unusual novel megastigmane sulfate from *P*. *luxurians*, which is here termed blumenol C sulfate (**1**). The occurrence of this compound is discussed in context to the peculiar habitat of the studied plant: the iron-oxyhydroxysulfate depositing Río Sucio (Figure 1b) [28]. Furthermore, we assessed possible antioxidative potentials and antimicrobial activities of this compound as well as the potential effects of the sulfate group in comparison to those of related compounds devoid of a sulfate group. In this regard, the sulfur and sulfate content of the studied *P*. *luxurians* and closely related species was also taken into account.

## 2. Results and Discussion

### 2.1. Isolation and Structure Elucidation of Compound 1

Compound **1** was isolated from the methanolic leaf extract from *P*. *luxurians* after employing various chromatographic separation techniques (Section 3.4). Analysis by HR-ESI-TOF-MS in negative mode exhibited a *m*/*z* 289.1112 for [M-H]^-^. The identified negatively charged molecule ion C_13_H_21_O_5_S^-^ corresponds to the calculated *m*/*z* 289.1115. In the positive mode a *m*/*z* 335.0899 for [M-H+2Na]^+^ (calcd *m*/*z* 335.0899), and 313.1090 for [M+Na]^+^ (calcd *m*/*z* 313.1080) were detected. All data are in good agreement with a molecular formula C_13_H_22_O_5_S. In the NMR spectra four methyl-, three methylene-, three methine groups and three quaternary carbon atoms were identified. Consequently, an acidic proton is present in the structure. Furthermore, 1D and 2D NMR data indicated a megastigmane-type skeleton.

Firstly, a cyclohexenone moiety was identified. A conjugated ketone group was characterized by its chemical shift of δC 202.4 ppm as well as a predominant absorbance of ν_C=O_ 1650 cm^−1^ in the infrared spectrum. The position of the olefinic proton H_1_-4 (δH 5.81 ppm, δC 125.4 ppm) was defined by its singlet in ^1^H NMR as well as ^3^*J*_H-C_ coupling with C-2, C-6, C-11 in HMBC. Additional ^2,3^*J*_H-C_ heteronuclear coupling of protons and carbon atoms in the three methyl groups in positions 11, 12 and 13 led to further characterization of the six membered ring structure. The methyl group in position 11 was located, due to its higher chemical shift of δH 2.05 ppm and ^2,3^*J*_H-C_ coupling with C-4 and C-5. The singlets of H_3_-12 and H_3_-13 exhibited ^3^*J*_H-C_ coupling with each other and with C-1, C-2 as well as C-6. Consequently, both methyl groups were linked to the same quaternary C-1 (δC 37.3 ppm). All chemical shifts in ^1^H NMR and ^13^C NMR for the cyclohexenone moiety were in good agreement to related structural data in previous reports [29,30].

Further investigation of the NMR-spectroscopic data indicated a second moiety, namely a *sec*-butyl residue. A methylene group in position 7 was localized due to ^2^*J*_H-C_ coupling with H_1_-6 of the six membered ring and H_2_-8 of the alkyl residue. In addition, ^3^*J*_H-H_ coupling between H_1_-9 and H_2_-8 as well as H_3_-10 of the terminal methyl group in 2D-COSY defined the position of all 13 carbons of the megastigmane.

Ultimately, a sulfate group was identified to be linked to C-9. The chemical shift of the proton and carbon in position 9 (δH 4.40 ppm, δC 77.1 ppm, CD_3_OD) were significantly shifted to the lower field compared to structurally similar megastigmanes, e.g., blumenol C with a hydroxyl group (δH 3.70 ppm, δC 68.9 ppm, CD_3_OD) [30] or blumenol C glucoside bearing a β-D-glucoside (δH 3.82 ppm, δC 77.7 ppm, CD_3_OD) [29] in the respective position. However, the chemical shifts of position 9 strongly correlated to the ones of a synthetized megastigmane, namely α-ionol sulfate (δH 4.25 ppm, δC 72.0 ppm, DMSO) with a comparable structural moiety [31]. Apart from that, IR spectroscopic data indicated a sulfate group, due to a strong absorption band at ν_S=O_ 1214 cm^−1^ and 1241 cm^−1^ (Supplementary Materials: Figure S9), which is characteristic to sulfone esters [32]. In comparison to blumenol C with a hydroxy group in the same position, these absorption bands were not detectable [30]. The IR spectrum (Figure S9) confirms other structural details of compound **1**. The ν_aCH3_ 2963 cm^−1^ and δ_CH3_ 1440 cm^−1^ confirm the CH_3_ group as well as the ν_aCH2_ 2938 cm^−1^ and δ_CH2_ 1379 cm^−1^ indicate the methylene groups. The δ_C=C_ 781 cm^−1^ further indicates the C=C double bond in the present cyclohexenone. In addition, the ν_C=O_ 1650 cm^−1^ confirms the carbonyl group and ν_C−O_ 1044 cm^−1^ indicates the C-O bond. Additionally, the molecular ion peak in the mass spectrum (negative mode) contained an isotope peak (A + 2) with a relative height of about 5.3%, which correlates to the ^34^S isotope and five ^18^O isotopes present in compound **1** (Figure S8).

The relative stereochemistry was partly determinable by a 2D-NOESY experiment. A cross signal between H_1_-6 and H_1_-9 was detected, but H_1_-6 showed NOEs to both H_3_-12 and H_3_-13. However, H_2_-7 had two NOEs only to H_3_-13 indicating the spatial closeness. Hence, ultimately, the distinction between positions 12 and 13 was accomplished by comparison with reported data of similar compounds [30].

**Figure 2:**
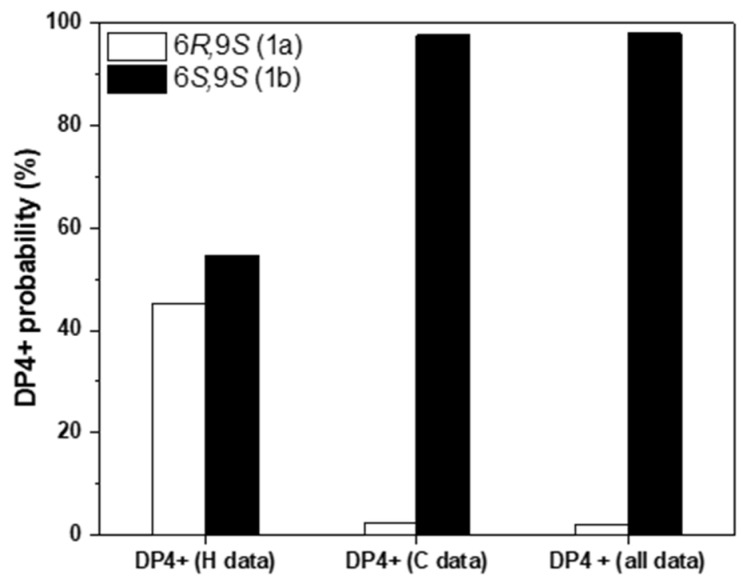
qccNMR coupled with DP4+ probability analysis of compound **1** for the stereo isomers **1a** (9*R*,9*S*, white) and **1b** (6*S*,9*S*, black).

To assign the relative configurations of C-6 and C-9 in the flexible alkyl chain, quantum chemical calculations of the NMR data (qccNMR) of two possible enantiomers of **1a** (6*R*,9*S*), and **1b** (6*S*,9*S*) were performed. The two possible configurations were subjected to a strict conformational screening procedure and subsequently the NMR chemical shifts were calculated at mPW1PW91/6-31+G(d,p)//M06-2X/Def2SVP level of theory with PCM solvent model in methanol. The DP4+ analysis also identified (6*S*,9*S*)-**1b** as the most likely structure of **1** with 98.01% DP4+ probability (all data) (Figure 2, Tables S1–S3).

**Figure 3:**
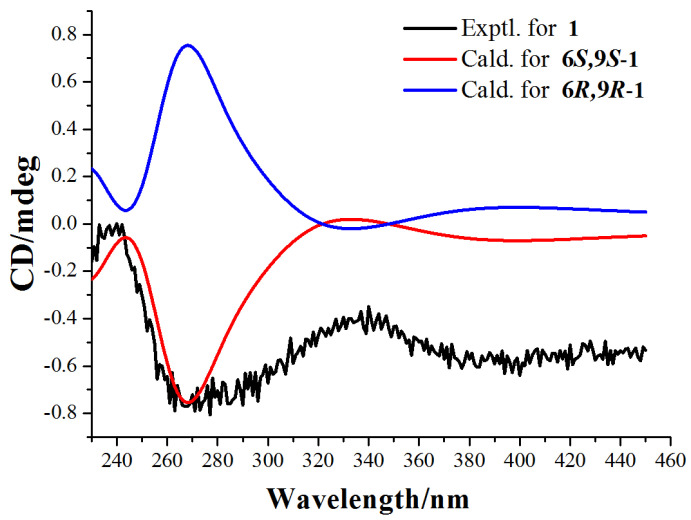
Experimental ECD spectra and calculated ECD spectra of compound **1** at the M06-2X/Def2SVP level in methanol.

Finally, the absolute configuration of compound **1** was assigned as 6*S*,9*S* by comparison of calculated and experimental ECD data. The experimental ECD spectrum of compound **1** is in good agreement to the calculated spectrum of (6*S*,9*S*)-**1**, but does not correlate to the (6*R*,9*R*)-**1** enantiomer (Figure 3 and Figure S11). Therefore, the absolute configuration of compound **1** was defined as 6*S*,9*S*.

**Figure 4:**
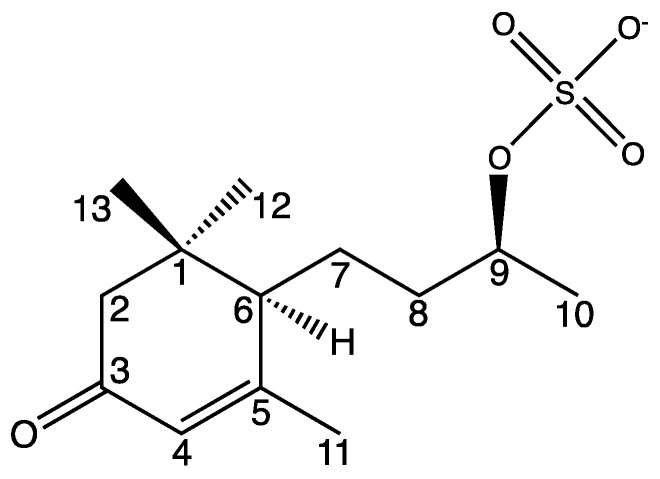
Structure of blumenol C sulfate (**1**). Numbering of carbon atoms is in accordance to Yan et al. [30] as well to numbering in Table 1.

The newly described compound (6*R*,9*R*)-**1** is named blumenol C sulfate (**1**) and is depicted in Figure 4. Naming as well as the numbering of the carbon atoms was based on the known blumenol C [25] and its derivative blumenol C glucoside [24]. A list of the NMR data of blumenol C sulfate (**1**) is presented in Table 1 and relevant spectra are provided in the Supplementary Material (Figures S1–S7).

### 2.2. Possible Biosynthesis

Blumenol C is quite likely the last key intermediate in the biosynthetic pathway leading to compound **1**. Its biosynthesis can be traced back to carotenoids that occur in all photosynthetic organisms. In the carotenoid metabolism in plants apocarotenoids arise from oxidative and enzyme catalyzed cleavages of carotenoids. As carotenoids bear many double bonds, a high number of possible apocarotenoids result as cleavage products. Those are considered as crucial compounds for various biological processes in plants [33]. One important group of apocarotenoids are megastigmanes with their typical C_13_ skeleton of, e.g., ionones, damascones or blumenols [7]. Three main representatives of blumenols, namely blumenol A, B, C are described. They all contain the same ring structure with an α,β-unsaturated keto group. Blumenol A—for which vomifoliol is the older name—differs from blumenol B only in the C-7/C-8 double bond. The carbon skeleton of blumenol C is equivalent to those of blumenol B. However, it has one hydroxyl group less in position C-6 [7,34]. Three blumenol derivatives have been reported to play various important roles for plants [34].

Blumenol C often occurs unsubstituted as well as glycosylated. In particular, blumenol C glucoside was frequently found in plants [29]. Apart from that, other megastigmane glycosides with different mono- and disaccharides have been reported [35]. In addition to those derivatives, Aasen et al. described a blumenol C acetate in Greek tobacco (*Nicotiana tabacum* L.; Solanaceae) [36] and Deng et al. isolated a blumenol C isobutyrate from roots and rhizomes of *Nardostachys chinensis* Batal. (Caprifoliaceae) [37]. Other acyl esters of the hydroxyl function in position C-9 have not yet been reported as natural products. To the best of our knowledge, no esters of inorganic acids (e.g., phosphate or sulfate) are reported so far [38].

A detailed mechanism for the sulfation of the hydroxyl group cannot be proposed solely from the isolation of blumenol C sulfate (**1**). However, a spontaneous, non-enzyme-catalyzed esterification with free sulfate anions is very unlikely. Furthermore, a non-substrate-specific inversion of a sulfatase catalyzed hydrolysis [E.C. 3.1.6.-] is also implausible [39]. For both transformations, an extremely high concentration of free sulfate anions would be necessary, which was not detected in the dried leaf samples of the investigated *P. luxurians* (see Section 2.4).

Thus, a sulfotransferase catalyzed esterification [E.C. 2.8.2.-] of blumenol C to compound **1** is the most likely catalytic process for biosynthesis. Compound **1** is, however, not a macromolecule and it has not yet been described in metabolic processes in plants. Hence, the acting sulfotransferase is most likely water soluble and mainly functions for detoxification purposes in plants [40,41]. This assumption can be supported by a sulfur-rich environment, which might lead to elevated sulfate levels and consequently to salt stress in the studied individual (see Section 2.3). Such an abiotic stress can lead to metabolic changes even on the level of specialized metabolism, as described for another plant species [42].

### 2.3. Sulfur Enriched Hydro- and Geochemical Environment

The sulfate ester group in compound **1** and the possible salt stress of the investigated plant imply an excessive supply of sulfur and sulfate. In this context two possible sources merit discussion. The soils in this part of the Braulio Carrillo National Park are derived from Pleistocene lava flows, and show a mean of 1.8 mg L^−1^ sulfate per liter of soil [43]. However, the plant material was collected at the banks of Río Sucio, which has a peculiar chemistry including an abundance of sulfur present as sulfate. The river has a brownish-yellowish color (Figure 1b), originates from the northern flank of Irazú volcano at about 3000 m, and is hence influenced by acid volcanic brines. Its confluence with the Río Honduras with its clear waters is a famous photo spot among tourists visiting the Braulio Carrillo National Park. However, only recently the Río Sucio—which translates to “dirty river”—was chemically studied: At 22 km downstream from its source, the river still shows a very high sulfate (502 ± 29 mg L^−1^) and iron content (5.20 ± 0.11 mg L^−1^) which leads to the dominance of the iron- and sulfur-oxidizing betaproteobacteria *Gallionella* OTU RS001 and *Acidithiobacillus ferrooxidans*. Their activities precipitate the nanocrystalline iron-oxyhydroxysulfate mineral schwertmannite, which ultimately leads to the yellow color and the name of the river (Figure 1b) [28].

By coincidence the material of *P. luxurians* studied here was collected at the exact sampling point of Arce-Rodríguez et al. [28] demonstrating an elevated sulfate content in the waters and deposits in the immediate vicinity. This suggests that the secondary metabolites of *P. luxurians* are indeed influenced by the local hydro- and geochemistry. Furthermore, it seems plausible the excess of sulfate is incorporated into a megastigmane leading to the novel blumenol C sulfate (**1**).

### 2.4. Elemental Analysis of the Leaves from P. luxurians

Organic elemental microanalysis is an established tool not only capable of characterizing the total elemental composition of pure substances but also useful for biogenic materials like dried leaves. An amount of only 1–2 mg of sample is sufficient for a single analysis of C, H, N, O or S, the latter being of particular interest in this study. The method determines the main constituents with a maximum uncertainty of ±0.3% (*w*/*w*) down to a LOQ of 0.05% (*w*/*w*) [44].

Triplicate analysis of ground and dried leaves confirmed an increased content of sulfur in *P. luxurians* leaves found in the sulfur enriched environment of the Braulio Carillo National Park (Sample A), from where blumenol C sulfate (**1**) was isolated. Another *P. luxurians* plant from the Volcán Tenorio National Park (Sample B) as well as three individuals of the putative close relative *Palicourea berteroana* (DC.) Borhidi (Sample C–E), collected from other low sulfur locations served as comparison samples (Table 2 and Table S6). The total elemental composition was complemented by ion analysis of aqueous extracts from all samples. Sulfate and chloride were detected at elevated concentrations indicating the relation of S-content obtained by sulfate supply from the environment. The results presented in Table 2 show a variance significantly higher than the typical uncertainty of the methods, due to the natural inhomogeneity of the investigated biogenic material.

The highest sulfur content (0.750%) was found in sample A from which compound **1** was isolated. It is noteworthy that this sulfur content was mainly detected as sulfur bound in sulfate ions (0.566%). A clearly reduced sulfur content (0.411%) was detected in sample B, collected in another region of Costa Rica about 50 m away from Río Celeste. This sulfur content was also detected mainly as free sulfate (0.313%).

The sulfate level measured for Río Celeste is only approximately 20% of those in Río Sucio [28,45]. However, at a hydrothermal spring in this river, the sulfate content is ca. 50% of that found in Río Sucio [28,46]. These results of the sulfur and sulfate analysis of the two directly comparable *P. luxurians* accessions indicate an increased sulfate uptake in a sulfur rich environment compared to the environment with comparably lower sulfur content. An occurrence of compound **1** in sample B can neither be confirmed nor ruled out due to small sample amount. Therefore, it should be clarified in future investigations whether *P. luxurians* and related species tend to biosynthesize sulfonic acid esters under increased SO_4_^2−^ stress.

Apart from that, lower total sulfur levels were determined in the three targeted *P. berteroana* leaves (sample C: 0.535%, sample D: 0.555%, sample E: 0.388%) when compared to sample A. However, for all three individuals the proportion of sulfur accumulated as free-sulfate was significant lower comparted to those in samples A and B, namely 0.125% for sample C and <0.05% for sample D and E. Hence, in *P. luxurians* there is a higher proportion of sulfur present as free sulfate ions compared to those in *P. berteroana*.

These results indicate an increased salt stress by SO_4_^2−^ ions in particular for the individual from which compound **1** was isolated. Thus, a change of the metabolism in this plant to achieve a sulfate detoxification cannot be excluded.

Both samples of *P*. *luxurians* leaves (samples A and B) exhibited a clearly reduced nitrogen content in comparison to the samples C, D, E, and sample A also shows a significant high chloride content (Table 2). Whether this rather low nitrogen content is characteristic for *P*. *luxurians* cannot be stated since comparable data is not available. Deeper discussion of these data would require further information on leaf nitrogen content from more individuals of the genus *Palicourea*.

### 2.5. Bioactivity

Regardless, whether blumenol C sulfate (**1**) is formed due to abiotic stress by SO_4_^2−^ ions in the investigated individual, it may exhibit bioactivities, which could be of additional benefit for the plant. Therefore, we examined the antimicrobial activity against *B. subtilis, E. coli* and *E. coli* ampR, as well as the antioxidant assays FRAP (ferric reducing antioxidant power) and ABTS (2,2-azinobis-(3-ethylbenzothiazoline-6-sulfonate)) of compound **1**. We compared the results to those of structurally related megastigmanes blumenol C glucoside from *Faramea tamberlikiana* subsp. *sessifolia* (P. H. Allen) C. M. Taylor (Rubiaceae) and vomifoliol isolated from *Palicourea adusta* (Figures S12 and S13; Tables S4 and S5).

**Figure 5:**
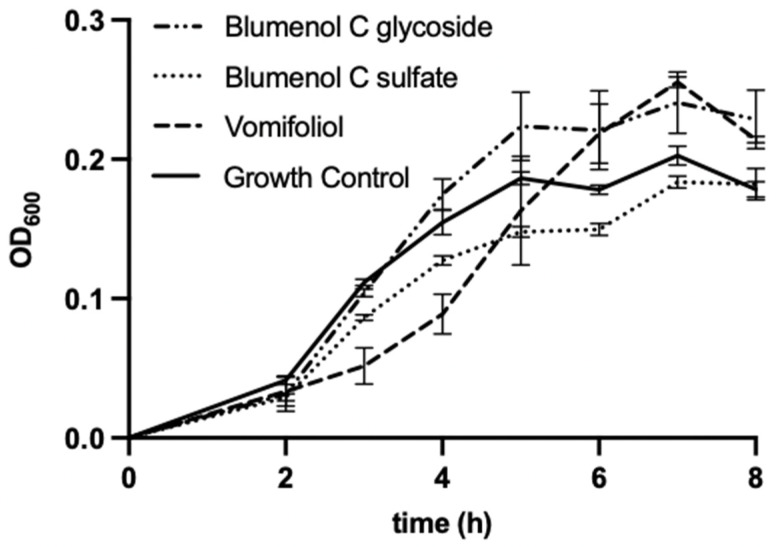
Microdilution assay of blumenol C sulfate (**1**), blumenol C glucoside and vomifoliol (=blumenol A), each 200 µg mL^−1^, against *B. subtilis*. Data shows means of three experiments ± SD.

The bacterial strains *E. coli* and *B. subtilis* were chosen as test-organisms as they were also used by Kuene et al. [16] in their study with vomifoliol which allows the comparison of results. The additional use of an ampicillin-resistant *E. coli* strain served to indicate potential antimicrobial activity of the compounds against antibiotic-resistant bacteria. Blumenol C sulfate (**1**), as well as vomifoliol, showed weak antibacterial effects against *B. subtilis* when applied at concentration of 200 µg mL^−1^ (Figure 5), however for blumenol C glucoside no antibacterial effects can be reported at the tested concentration. Compound **1** led to a moderate decrease of the growth of *B. subtilis* up to 7 h of incubation. Vomifoliol reduced the growth of *B. subtilis* strongly up to 4 h of incubation compared to the growth control. However, with increasing incubation time, the antibacterial effect diminishes.

Against *E. coli* and *E. coli* ampR no significant antibacterial activity of the three investigated megastigmanes can be observed. This difference in antimicrobial efficacy against *B. subtilis* and *E. coli* is in partial agreement with the results reported by Kuete et al. [16], who reported moderate antimicrobial activity of vomifoliol from leaves of *Treculia* species against both bacterial strains.

In addition, the FRAP and ATBS assays for the three megastigmanes at a concentration of 100 µg mL^−1^ were performed. However, no antioxidant activity can be reported from both assays. This is in agreement with earlier reports, which indicate that several megastigmanes do not act as antioxidants [19,47], but is in contrast to other reports [4,17]. The missing antioxidant activity is, however, not totally unexpected since the tested megastigmanes are lacking certain structural features usually responsible for radical scavenger activities [48,49].

The conducted experiments revealed that blumenol C sulfate (**1**) exhibits moderate antibacterial effects but no antioxidant potential. However, the effects are comparable to those of other megastigmanes possessing similar structures. Thus, with respect to the investigated bioactivities no clear advantages seem to arise for *P. luxurians* from an accumulation of blumenol C sulfate (**1**) as specialized metabolite.

## 3. Materials and Methods

### 3.1. Plant Material

#### 3.1.1. Plant Material Used for Isolation

The plant material of *Palicourea luxurians* (sample A in Table 2) was collected along the banks of the Río Sucio in the Braulio Carrillo National Park, Costa Rica, just after its confluence with the Río Honduras, ca. at 10°08′54″ N 83°56′52″ W. A corresponding voucher specimen (*A. Berger & J. Schinnerl 2069*; WU 0103854) is stored at the Herbarium of the University of Vienna (WU).

*Palicourea adusta* was collected at Barva Vulcano, Braulio Carrillo National Park, Costa Rica, and corresponding voucher specimens (*A. Berger AB 10021002*) are deposited in the Herbaria of the Missouri Botanical Garden (MO) (MO-2531331) and WU (WU 0067360, 0067361, 0067362, 0067363). *Faramea* *tamberlikiana* subsp. *sessifolia* was collected near the Field Station La Gamba, Piedras Blancas National Park, Costa Rica. A voucher specimen (*A. Berger 1862*) is deposited at WU (WU 0099997). Digital images and additional collection data may be found at the international herbarium database system JACQ (https://www.jacq.org, accessed on 12 October 2022). Collection of plant material was kindly permitted by the Costa Rican Ministry of Ambient and Energy under resolutions 191-2009-SINAC and R-055-2016-OT-CONAGEBIO.

#### 3.1.2. Plant Material Used for Elemental Analysis

Sample A: See Section 3.1.1.; sample B: *P*. *luxurians* from Volcán Tenorio National Park, Costa Rica (WU 0100060); C: *P. berteroana* from Braulio Carrillo National Park, Costa Rica (WU 0088354); D: *P*. *berteroana* from near Wilson Botanical Garden, Costa Rica (WU 0088353); E: *P*. *berteroana* from Gandoca—Manzanillo Wildlife Refuge, Costa Rica (WU 0088352).

### 3.2. Extraction and Isolation

The ground air-dried leaves of *Palicourea luxurians* (113.5 g) were exhaustively extracted with methanol (MeOH) at room temperature (three times 2 d) yielding 23.7 g residue. Suspending of this residue in water and subsequent partitioning between petrol ether, chloroform, ethyl acetate and *n*-butanol yielded 5.9 g *n*-butanol phase. Column chromatography (CC) of 520 mg aliquot of this butanol phase over silica gel 60, 40–60 µm particle size afforded 79.7 mg of impure **1**. Further purification of this fraction via CC over Sephadex LH20 eluted with MeOH afforded 5.0 mg of purified compound **1**. The details of the extraction and isolation procedure of vomifoliol and blumenol C glucoside are provided in the supplementary material.

Blumenol C sulfate (**1**): White amorphous powder; C_13_H_22_O_5_S; MS, *m/z*: [M − H + 2Na]^+^ 335.0899 (calcd for [M − H + 2Na]^+^ 335.0899), [M + Na]^+^ 313.1090 (calcd for [M + Na]^+^ 313.1080), [M − H]^−^ 289.1112 (calcd for [M − H]^−^ 289.1115); UV_max, MeOH_, 244 nm (ε 3.79 L mol^−1^ cm^−1^); IR, cm^−1^: 3455, 2963, 2938, 2874, 1650, 1440, 1379, 1241, 1214, 1081, 1044, 933, 908, 781, 627 and 586. Optical rotation: [α]_D_^20^ (*c* 2.6 mg mL^−^^1^, CH_3_OH) = +48.5°. For NMR spectroscopic data see Table 1.

### 3.3. Chromatographic Purification

HPLC analyses were performed on Agilent 1100 series with UV-diode array detection using a Hypersil BDS-C18 (250 × 4.6 mm, 5 μm particle size; Agilent, St. Clara, CA, USA) column at a flow rate of 1.0 mL min^−1^ and an injection volume of 10 µL. An aqueous solution containing 10 mM ammonium acetate (A) and methanol (MeOH) (B) were used as eluents, the following gradient was used: from 40–90% B in A within 12 min, from 90–100% B in A within 0.1 min and 100% B was kept for 5.9 min. The wavelength of detection was set at 230 nm (reference wavelength 360 nm). TLC analyses were done on silica gel 60 F_254_ plates, layer thickness 0.2 mm (Merck, Kenilworth, NJ, USA) developed with CHCl_3_/MeOH 80:20 or with the organic phase of *n*-butanol/acetic acid/water 50:10:40. Size exclusion chromatography (SEC) was performed by using Sephadex LH-20 (GE Healthcare, Chicago, IL, USA) as stationary phase eluted with MeOH. Another stationary phase for column chromatography (CC) was silica gel 60 (Merck) with 40–63 μm particle size.

### 3.4. Spectroscopic and Spectrometric Measurements

For NMR spectroscopic measurements compound **1** was dissolved in CD_3_OD (~3.0 mg in 0.7 mL) and transferred into 5 mm high precision NMR sample tubes. All spectra have been measured on a Bruker Avance III 600 at 600.13 MHz (^1^H) or 150.61 MHz (^13^C) and processed using the Topspin 3.5 software. 1D spectra were recorded by acquisition of 32 k data points and after zero filling to 64 k data points, and Fourier transformation spectra were processed with a range of 7200 Hz (^1^H) and 32,000 Hz (^13^C), respectively. To determine the 2D COSY, TOCSY, NOESY, HSQC, and HMBC spectra, 128 experiments with 2048 data points each were recorded and Fourier-transformed to 2D-spectra with a range of 6000 Hz and 32,000 Hz for ^1^H and ^13^C NMR, respectively. Measurement temperature was 298 ± 0.05 K. Residual, not fully deuterated CD_3_OD was used as internal standard for ^1^H (δH 3.34) and CD_3_OD for ^13^C (δC 49.0) measurements. CH_3_, CH_2_, CH and C_q_ are indicated by the multiplicities (q, t, d, s), respectively, which indicate the signal form, as if the ^13^C NMR measurements had been taken without proton broadband decoupling.

The IR spectrum was recorded using a Cary 630 FTIR instrument (Agilent) with a single reflection diamond ATR-unit and a KBr-optic. Four single spectra were recorded after evaporating a 1.0 µL drop of methanolic solution of the sample (32 scans at a resolution of 2 cm^−1^). The average spectrum is presented in Figure S9.

The molar extinction coefficient was determined based on measurements on a Specord 205 Photometer (Analytic Jena, Jena, Germany) using a 10 mm cuvette.

Optical rotation was measured at the sodium D line using a 100 mm path length cell on a PerkinElmer Automatic Polarimeter 241.

Far-UV circular dichroism (CD) spectra were recorded at 25 °C on an Applied Photophysics Chirascan Plus system (SurreyK). Compound **1** was diluted in acetonitrile to a final concentration of 50 mM. CD spectra were collected at a scan speed of 20 nm min^−1^ at 1 nm bandwidth and response time of 4 s. All spectra were recorded in a 0.1 cm cuvette between 230 and 460 nm. The data shown are the average of ten recorded spectra.

Mass spectra were recorded on a high-resolution time-of-flight (HR-TOF) mass spectrometer (maXis, Bruker Daltonics, Billerica, MA, USA) by direct infusion electrospray ionization (ESI) in positive ionization mode (mass accuracy ± 5 ppm) as well as in negative mode (mass accuracy ± 10 ppm). HR-TOF MS measurements have been performed within the selected mass range of *m*/*z* 100–2500. ESI was made by capillary voltage of 4 kV to maintain a (capillary) current between 30–50 nA. Nitrogen temperature was maintained at 180 °C using a flow rate of 4.0 L min^−1^ and the N_2_ nebulizer gas pressure at 0.3 bar.

### 3.5. Quantum Chemical Calculation Based on NMR and CD Data

Conformation search based on molecular mechanics with MMFF force fields were performed for the diastereomers **1a** and **1b**. This resulted in 6 and 5 stable conformers with populations higher than 1%, which are shown Figure S10. All these conformers were further optimized by the density functional theory method at the M06-2X/Def2SVP level by Gaussian 09 program package with g09 default keyword. Gauge Independent Atomic Orbital (GIAO) calculations of their ^1^H and ^13^C NMR chemical shifts were performed using density functional theory (DFT) at the mPW1PW91/6-311+G(d,p) level with the PCM model in methanol. The calculated NMR data of these conformers were averaged according to the Boltzmann distribution theory and their relative Gibbs free energy (Tables S1–S3). The ^1^H and ^13^C NMR chemical shifts for TMS were also calculated by the same procedures and used as the reference. After calculation, the experimental and calculated data were evaluated by the improved probability DP4+ method (Tables S1 and S2).

Conformation searches based on molecular mechanics with MMFF force fields were performed for 6*S*,9*S*-**1** and 6*R*,9*R*-**1** (Figure S11) and gave five stable conformers with populations higher than 1%. All these conformers were further optimized by the density functional theory method at the M06-2X/Def2SVP level by Gaussian 09 program package with g09 default keyword. The ECD were calculated using density functional theory (TDDFT) at M06-2X/Def2SVP level in methanol with IEFPCM model (Figure 3). The calculated ECD curves were all generated using SpecDis 1.71 with *σ* = 0.30 eV.

### 3.6. Elemental Analysis

C/H/N/S-analysis was done using the EA 3000 CHNS-O elemental analyzer (Eurovector, Pavia, Italy). The ground leaves of each plant were ground and dried *in vacuo* in an amount of ca. 50 mg prior analysis. Accurately weighed 1.0–2.0 mg of each sample was transferred into tin capsules and analyzed. The instrument uses rapid flash combustion applying a pressure pulse of oxygen for digestion. The oxidation process is completed using WO_3_ on Al_2_O_3_ as catalyst. Excess oxygen is trapped on elemental copper acting as a reducing reagent to realize Dumas-nitrogen determination. The elements C, H, N and S were quantified, due to the stoichiometric conversion into N_2_, CO_2_, H_2_O and SO_2_. These gaseous products were analyzed using an online GC-system and a thermal conductivity detector.

Blank values are determined using empty tin vials. Calibration was done using National Institute of Standards and Technology (NIST) referenced certified standards for elemental analysis [sulphanilamide, 2,5-bis-(5-*t*-butyl-2-benzo-oxazol-2-yl)-thiophenone (BBOT)].

Oxygen determination uses the high temperature pyrolysis system at 1480 °C (HT 1500). Due to their low oxygen blank, silver capsules are used to weigh and introduce the samples. The samples are reduced at granulated carbon in high purity helium that serves as a carrier gas. The above-mentioned EA 3000 serves as GC and detection system.

Calibration was performed by using empty silver vials for blanks and certified standard reference material (acetanilide, l-cystine and benzoic acid; Sigma Aldrich, St. Louis, MO, USA) for calibration. Carbon monoxide is the analytical species stoichiometrically generated from oxygen in the sample. The analysis of ionic species was done in aqueous extracts from 1.0–2.5 mg sample in 25.0 mL of Milli-Q water. After filtration through a 0.4 µm membrane filter the solution was analyzed by a 7100 CE system (Agilent) coupled to a conductivity detector (TraceDec) using a Goods-buffer solution (CHES/Arg, pH = 9.1) with TTAOH as EOF modifier. External calibration was performed by standard mixed anion-solutions consisting of NaCl and Na_2_SO_4_ in analytical grade quality. For additional information about elemental analysis see supplementary material including Figures S14–S19 and Table S6.

### 3.7. Antioxidant Activity

The ferric reducing antioxidant power (FRAP) is based on the reduction of Fe^3+^ to Fe^2+^ by the antioxidant compound. A colored complex with 2,4,6-tripyridyl-*s*-triazine in acetate buffer with an absorption maximum at 593 nm is formed. The working solution was prepared with 25 mL of 0.3 M acetate buffer (pH 3.6), 2.5 mL of 10 mM 2,4,6-tripyridyl-*s*-triazine in 40 mM HCl, and 2.5 mL 20 mM FeCl_3_·6H_2_O in deionized water [50,51]. The investigated compounds were dissolved in MeOH (100 mg mL^−1^) and 50 µL were mixed with 300 µL FRAP working solution and subsequently the absorbance was recorded after 30 min (593 nm).

The second method is based on the scavenging of the ABTS radical 2,2′-azino-bis(3-ethylbenzothiazoline-6-sulfonic acid). The radical cation was generated by reacting 3.5 mM ABTS with 1.2 mM K_2_S_2_O_8_ in H_2_O. Subsequently, the mixture was stored in darkness at room temperature for 12–16 h and then diluted with EtOH to a working solution (absorbance of 0.700 at 734 nm) before used. After the addition of ABTS working solution (300 µL) to the samples (50 μL), the decrease of the absorbance was recorded after 30 min [51,52,53].

For both assays the absorbance was recorded on a SPECTROstar Nano absorbance microplate reader (BMG LABTECH) and the calibration was carried out with ascorbic acid (Sigma Aldrich).

### 3.8. Anti-Microbial Activity

Broth Microdilution Assay was performed according to the guidelines of the Clinical and Laboratory Standard Institute (CLSI) with minor changes [54]. *Escherichia coli* Top10 (Invitrogen, part of Life Technologies, Paisley, UK), *Bacillus subtilis* (Lab collection of Angela Witte, University of Vienna), and *E. coli* Top10 transformed with pUC18 (Thermo Scientific, part of Thermo Fisher Scientific Inc., Waltham, MA, USA) providing ampicillin resistance (further called *E. coli* ampR) were used as test organisms to perform the assay. Bacterial suspensions were prepared by inoculating fresh Mueller-Hinton Broth (Sigma-Aldrich, Co., St. Louis, MO, USA; 70192-500G) with an overnight culture (ONC) of the corresponding bacteria to an OD_600_ of 0.05. For *E. coli* ampR, ampicillin was added to the medium to a final concentration of 100 µg mL^−1^. The compounds were dissolved in methanol (MeOH) to a concentration of 1.0 mg mL^−1^ and pure MeOH was used as negative control (further referred to as “growth control”). The methanolic compounds were applied to 96-well plates in three replicates. MeOH was evaporated to complete dryness and 100 µL of bacterial suspension was applied, and the dried compound was resuspended with this bacterial suspension by pipetting up and down. This procedure resulted in a total concentration of extract in the reaction of 100 µg mL^−1^ and 200 µg mL^−1^, respectively. The microdilution assay was incubated for 8 h at 37 °C. OD_600_ was measured each hour using a plate reader (GloMax^®^ Multi Detection System, Promega™, Madison, WI, USA), starting at 0 h (directly after preparation of the assay) and finishing at 8 h of incubation. Evaluation of the data was performed using GraphPad Prism 9 (GraphPad Software LLC, San Diego, CA, USA).

## 4. Conclusions

Blumenol C sulfate (**1**) isolated herein from leaves of *P. luxurians* is the first described megastigmane directly esterified with a sulfate group. Its molecular structure and absolute stereochemistry were determined by various spectroscopic and spectrometric methods as well as quantum chemical calculations. Elemental analyses exhibited an increased level of sulfur, in particular in inorganic sulfate, in the leaves of this plant which is most likely caused by its sulfur rich geochemical environment. Bioactivity tests did not indicate advantages for the plant from accumulating the blumenol C sulfate (**1**), due to the moderate antimicrobial activity, which correlates to those of the other tested non-sulfated megastigmane, vomifoliol. Additionally, no antioxidant activity could be observed for compound **1** and two other the tested megastigmanes. The formation of the blumenol C sulfate (**1**) may contribute to the detoxification of SO_4_^2−^ in the plant tissue. The geochemical environment appears to produce salt stress for *P. luxurians*. This plant species does not seem to derive any further recognizable benefit from the additional elemental sulfur, at least in the case of the isolated metabolite blumenol C sulfate (**1**).

## Figures and Tables

**Table 1:** ^1^H and ^13^C NMR data of compound **1** in CD_3_OD measured on a 600 MHz NMR. Numbering of carbon atoms is in accordance to Figure 4.

pos.	δ ^1^H (ppm)	δ ^13^C (ppm)
1	-	37.3, s
2	1.97 (1H, m) 2.48 (1H, d, 16.9)	48.1, t
3	-	202.4, s
4	5.81 (1H, s)	125.4, d
5	-	169.9, s
6	1.99 (1H, m)	52.3, d
7	1.51 (1H, m) 2.02 (1H, m)	26.7, t
8	1.69 (2H, m)	37.7, t
9	4.40 (1H, ddq, 6.1, 6.1, 6.3)	77.1, d
10	1.32 (3H, d, 6.3)	21.2, q
11	2.05 (3H, d, 1.1)	24.9, q
12	1.01 (3H, s)	29.0, q
13	1.10 (3H, s)	27.5, q

**Table 2:** Results (% *w*/*w*) of the organic elemental microanalysis of dried *Palicourea luxurians* leaves, from which compound **1** was isolated (A), another plant of the same (B) and three plants of the related species *P. berteroana* (C–E) from other regions of Costa Rica. Plant A shows elevated content of S and SO_4_^2−^ compared to B–E.

Sample	C	H	N	S	O ^d^	Cl	Total	SO_4_^2−^	S from SO_4_^2−^
A	45.77 (*0.01*) ^b^	6.11 (*0.01*) ^b^	2.67 (*0.05*) ^b^	0.750 (*0.011*) ^b^	42.26	0.643 (*0.086*) ^a^	98.20	1.697 (*0.314*) ^a^	0.566
B	47.99 (*0.77*) ^b^	6.38 (*0.12*) ^b^	2.13 (*0.07*) ^b^	0.411 (*0.080*) ^b^	40.70	0.307 (*0.041*) ^a^	97.93	0.939 (*0.565*) ^a^	0.313
C	46.07 (*0.12*) ^b^	6.21 (*0.03*) ^b^	3.38 (*0.09*) ^b^	0.535 (*0.003*) ^b^	41.63	0.144 (*0.039*) ^c^	97.96	0.375 (*0.141*) ^c^	0.125
D	47.07 (*1.57*) ^b^	6.47 (*0.24*) ^b^	4.15 (*0.02*) ^b^	0.555 (*0.013*) ^b^	40.19	0.379 (*0.066*) ^c^	98.81	<0.05 ^c^	
E	47.22 (*0.22*) ^b^	6.46 (*0.06*) ^b^	3.36 (*0.03*) ^b^	0.388 (*0.004*) ^b^	39.44 (*0.19*) ^b^	0.314 (*0.011*) ^c^	97.19	<0.05 ^c^	

Results are the average of *N* individual analysis runs [^a^ (*N = 5*); ^b^ (*N = 3*); ^c^ (*N = 2*)]. The σ-values (in italics) are tabulated for all results of replicate measurements. ^d^ Oxygen values are mainly single results as the gas-purge in the auto-sampler caused a significant loss of remnant moisture. Only those values were evaluated that were recorded immediately after the placement of the sample to the analyzer.

## Data Availability

Data are available from the corresponding authors.

## Supplementary Materials

The following supporting information can be downloaded at: https://www.mdpi.com/article/10.3390/molecules27217284/s1, Figure S1: ^1^H NMR of **1** in CD_3_OD; Figure S2: ^13^C NMR [APT] of **1** in CD_3_OD; Figure S3: COSY of **1** in CD_3_OD; Figure S4: TOCSY of **1** in CD_3_OD; Figure S5: HSQC of **1** in CD_3_OD; Figure S6: HMBC of **1** in CD_3_OD; Figure S7: NOESY of **1** in CD_3_OD; Figure S8: HR-ESI-TOF-MS (negative mode) of **1**; Figure S9: FT IR spectrum of **1**; Figure S10: Two representative diastereomeric forms 6*R**9*S**-**1a** and 6*S**9*S**-**1b** used for conformation search based on molecular mechanics with MMFF force fields; Figure S11: Two representative enatiomeric forms (6*S*,9*S*)-**1** and (6*R*,9*R*)-**1** used for conformation search based on molecular mechanics with MMFF force fields. Figure S12: Structure of blumenol C glucoside; Figure S13: Structure of vomifoliol; Figure S14: C/H/N/S-Analysis on EA 3000: TCD-trace of a C/H/N/S-run on sample A; Figure S15: C/H/N/S-Analysis on EA3000: TCD-trace of a C/H/N/S-run on sample C; Figure S16: O-Analysis on EA3000 combined to the HT 1500 pyrolysis-system: TCD-trace of an analysis-run on sample A. Figure S17: O-Analysis on EA3000 combined to the HT 1500 pyrolysis-system: TCD-trace of an analysis-run on sample C. Figure S18: Comparison of two samples with a 10 µM mixed anion standard containing bromide, chloride and sulfate; Figure S19: Calibration for chloride and sulfate; Table S1: DP4+ analysis results of 6*R**9*S**-**1a** and 6*S**9*S**-**1b**; Table S2: Correlations between calculated (DP4+) and experimental ^1^H and ^13^C NMR chemical shifts of 6*R**9*S**-**1a** and 6*S**9*S**-**1b**; Table S3: M06-2X/Def2SVP optimized lowest energy 3D conformers and energy analysis for compound **1**; Table S4: ^1^H and ^13^C NMR spectroscopic data for blumenol C glucoside in CD_3_OD measured on a 600 MHz NMR; Table S5: ^1^H and ^13^C NMR of vomifoliol spectroscopic data in CD_3_OD measured on a 600 MHz NMR; Table S6: Detailed microchemical elemental analysis data for sample A–E.(Citation of [55]).

## Sample Availability

Samples of the compounds are available from the authors.
