# Supplementary material for: Phytochemistry Meets Geochemistry—Blumenol C Sulfate: A New Megastigmane Sulfate from Palicourea luxurians (Rubiaceae: Palicoureeae)
Source: Molecules. 2022 Oct 26;27(21):7284. doi: 10.3390/molecules27217284 (PMC9658315; doi:10.3390/molecules27217284)
Supplement: Supplementary file 1 [file molecules-27-07284-s001.zip › molecules-1960388-supplementary.pdf]

SUPPLEMENTARY MATERIAL

Phytochemistry meets geochemistry—Blumenol C sulfate: A new megastigmane sulfate from *Palicourea luxurians* (Rubiaceae: Palicoureeae)

Christoph Kornpointner [1,2], Nadine Hochenegger [2], Bao-Bao Shi [3], Andreas Berger [4], Johannes Theiner [5], Lothar Brecker [1,*] and Johann Schinnerl [4,*]

[1] Department of Organic Chemistry, University of Vienna, Währinger Strasse 38, A-1090 Vienna, Austria.

[2] Institute of Chemical, Environmental and Bioscience Engineering, Technische Universität Wien, Getreidemarkt 9/166, A-1060 Vienna, Austria.

[3] School of Pharmaceutical Sciences, South-Central MinZu University for Nationalities, Wuhan 430074, China

[4] Department of Botany and Biodiversity Research, University of Vienna, Rennweg 14, A-1030 Vienna, Austria.

[5] Microanalysis Services, University of Vienna, Währinger Strasse 42, A-1090 Vienna, Austria.

* Correspondence:
Johann Schinnerl (johann.schinnerl@univie.ac.at; Tel.: +43 1 4277 54072)
Lothar Brecker (lothar.brecker@univie.ac.at; Tel.: +43 1 4277 52131)

Table of Contents

Blumenol C sulfate (1) 4

Figure S1. ^{1}H NMR of **1** in CD_3OD 4

Figure S2. ^{13}C NMR [APT] of **1** in CD_3OD 5

Figure S3. COSY of **1** in CD_3OD 6

Figure S4. TOCSY of **1** in CD_3OD 7

Figure S5. HSQC of **1** in CD_3OD 8

Figure S6. HMBC of **1** in CD_3OD 9

Figure S7. NOESY of **1** in CD_3OD 10

Figure S8. HR-ESI-TOF-MS (negative mode) of **1** 11

Figure S9. FT IR spectrum of **1** 12

Figure S10. Two representative diastereomeric forms 6*R**9*S**-**1a** and 6*S**9*S**-**1b** used for conformation search based on molecular mechanics with MMFF force fields. 13

Table S1. DP4+ analysis results of 6*R**9*S**-**1a** and 6*S**9*S**-**1b** 13

Table S2. Correlations between calculated (DP4+) and experimental ^{1}H and ^{13}C NMR chemical shifts of 6*R**9*S**-**1a** and 6*S**9*S**-**1b** 14

Table S3. M06-2X/Def2SVP optimized lowest energy 3D conformers and energy analysis for compound **1** 15

Figure S11. Two representative enatiomeric forms (6*S*,9*S*)-**1** and (6*R*,9*R*)-**1** used for conformation search based on molecular mechanics with MMFF force fields 16

Blumenol C glucoside 17

Figure S12. Structure of blumenol C glucoside 17

Table S4. ^{1}H and ^{13}C NMR spectroscopic data for blumenol C glucoside in CD_3OD measured on a 600 MHz NMR 18

Vomifoliol (= blumenol A) 19

Figure S13. Structure of vomifoliol 19

Table S5. ^{1}H and ^{13}C NMR of vomifoliol spectroscopic data in CD_3OD measured on a 600 MHz NMR 19

Elemental analysis 20

Table S6. Detailed microchemical elemental analysis data for sample A–E 22

Figure S14. C/H/N/S-Analysis on EA 3000: TCD-trace of a C/H/N/S-run on sample A 23

Figure S15. C/H/N/S-Analysis on EA3000: TCD-trace of a C/H/N/S-run on sample C 23

Figure S16. O-Analysis on EA3000 combined to the HT 1500 pyrolysis-system: TCD-trace of an analysis-run on sample A 24

Figure S17. O-Analysis on EA3000 combined to the HT 1500 pyrolysis-system: TCD-trace of an analysis-run on sample C 24
Figure S18. Comparison of two samples with a 10 µM mixed anion standard containing bromide, chloride and sulfate 25
Figure S19. Calibration for chloride and sulfate 26

References 27

Blumenol C sulfate (1)

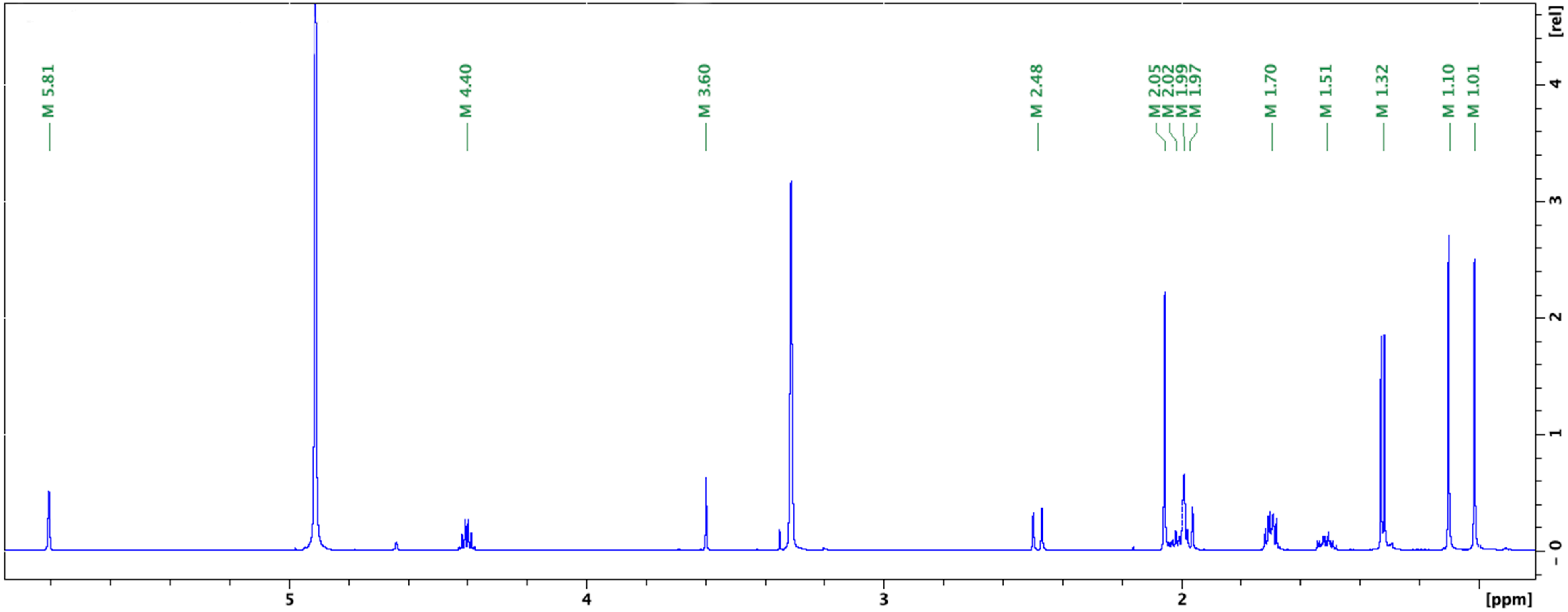

Figure S1. ^{1}H NMR of **1** in CD_3OD.

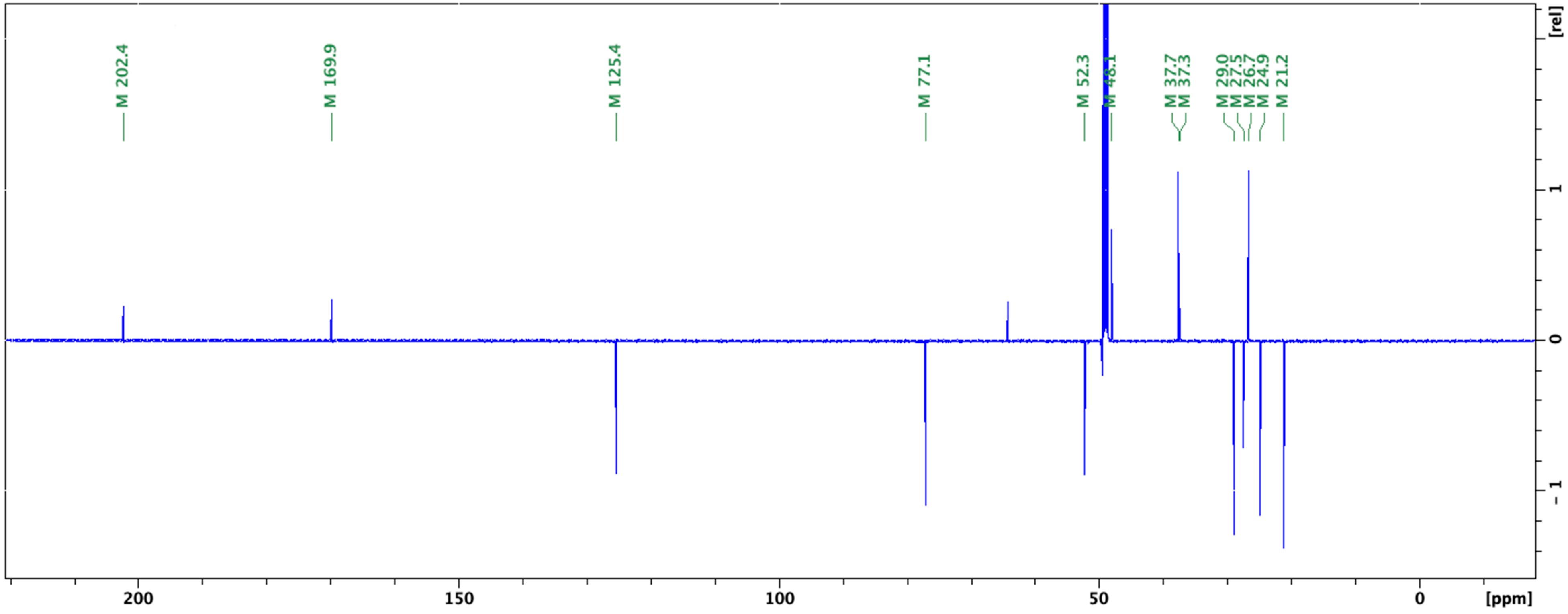

Figure S2. ^{13}C NMR [APT] of **1** in CD_3OD.

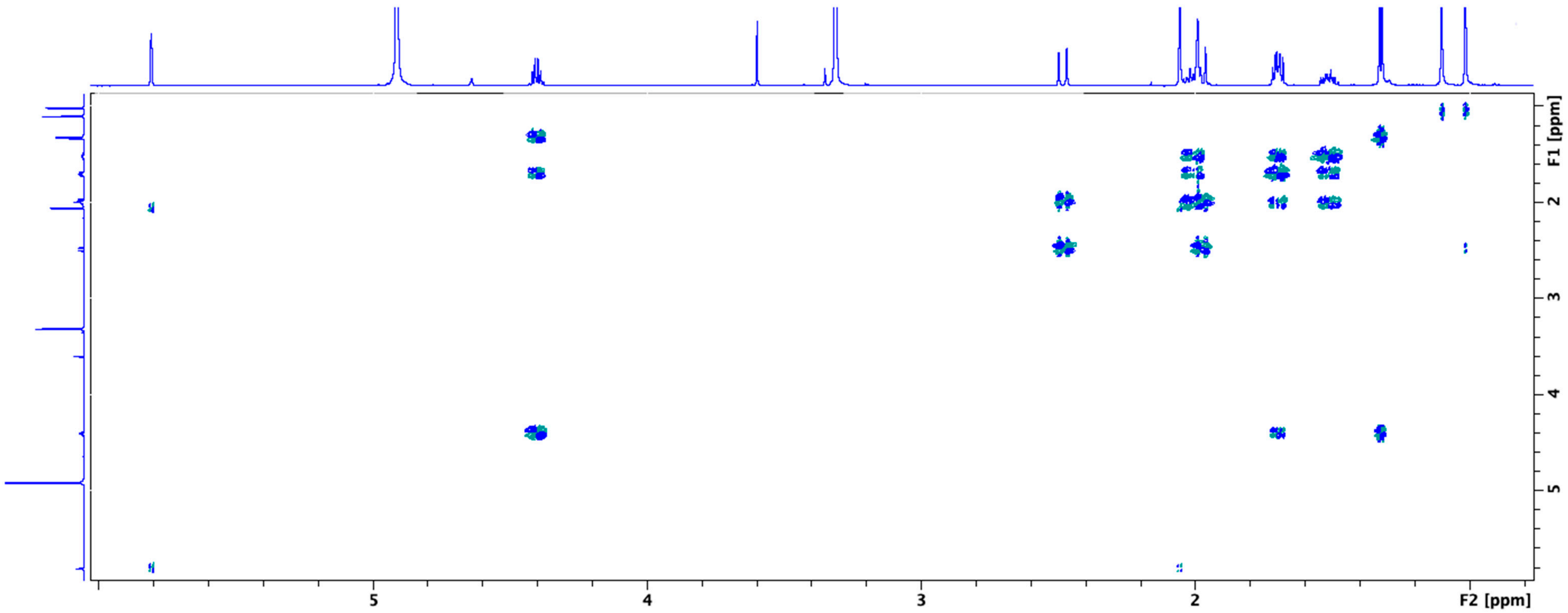

Figure S3. COSY of **1** in CD_3OD.

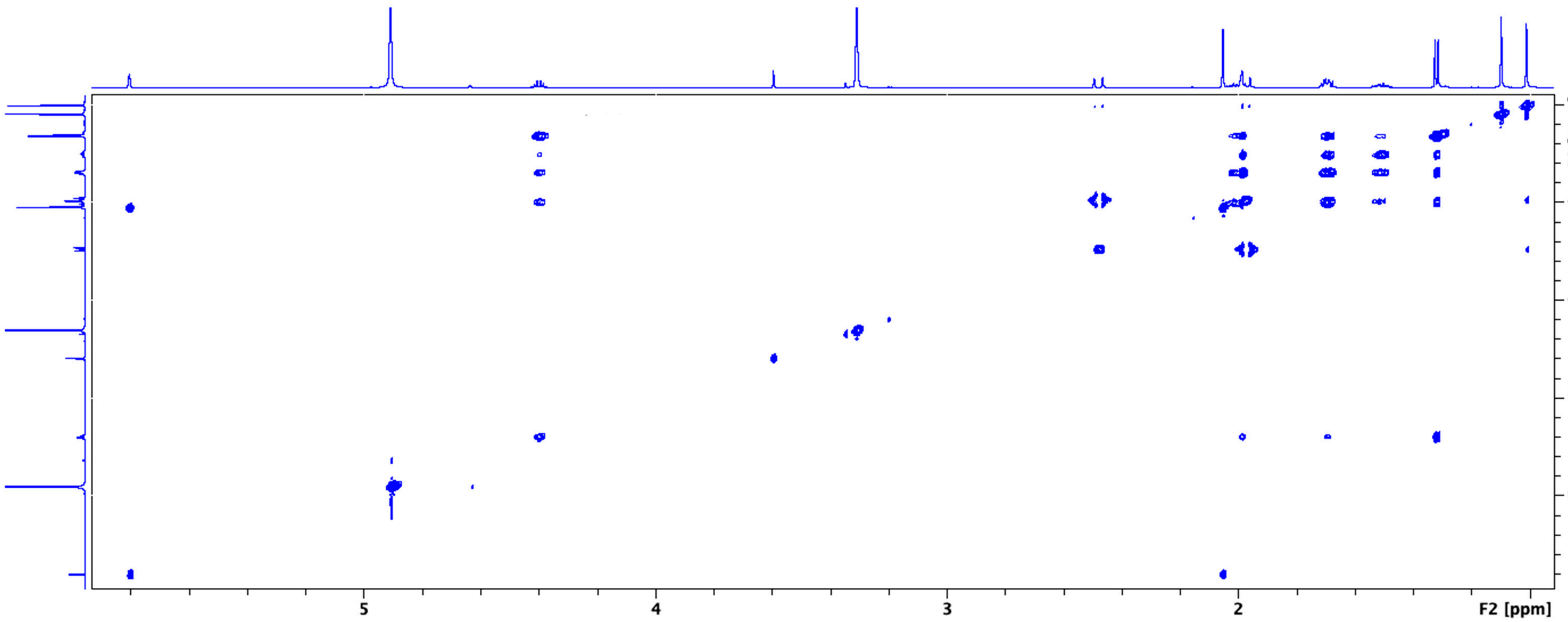

Figure S4. TOCSY of **1** in CD_3OD.

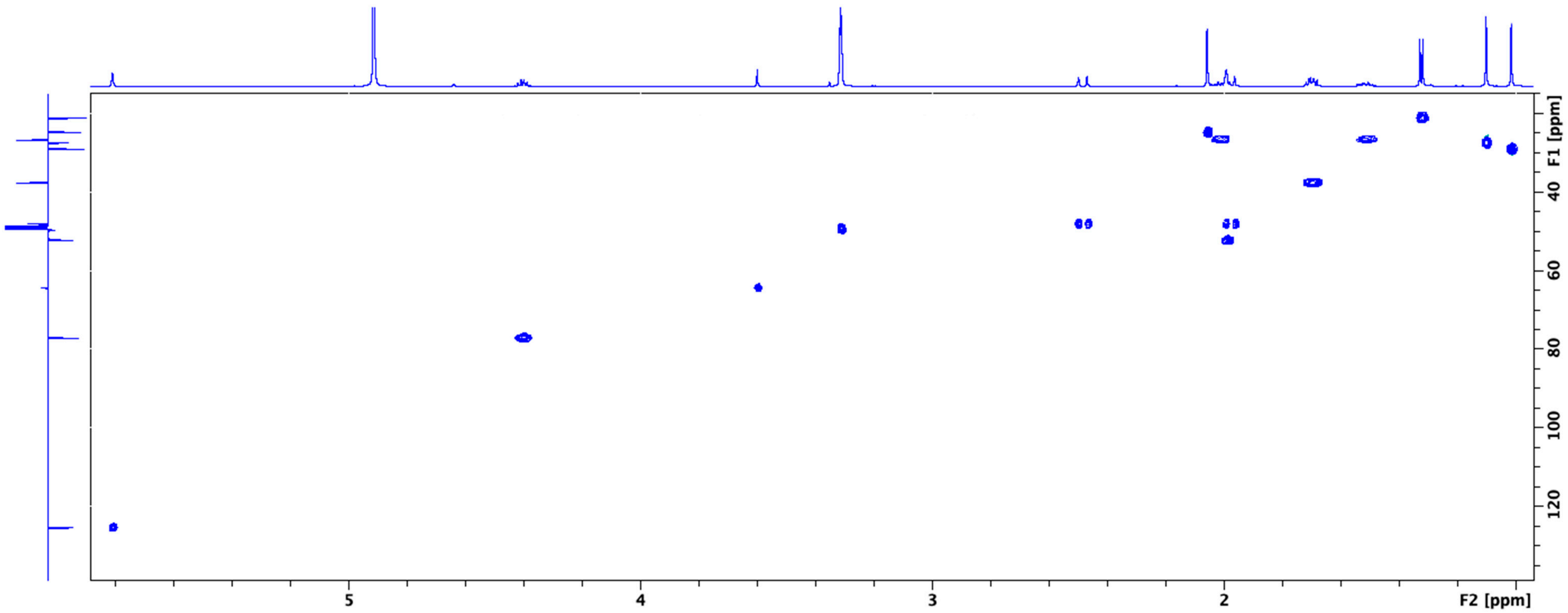

Figure S5. HSQC of **1** in CD_3OD.

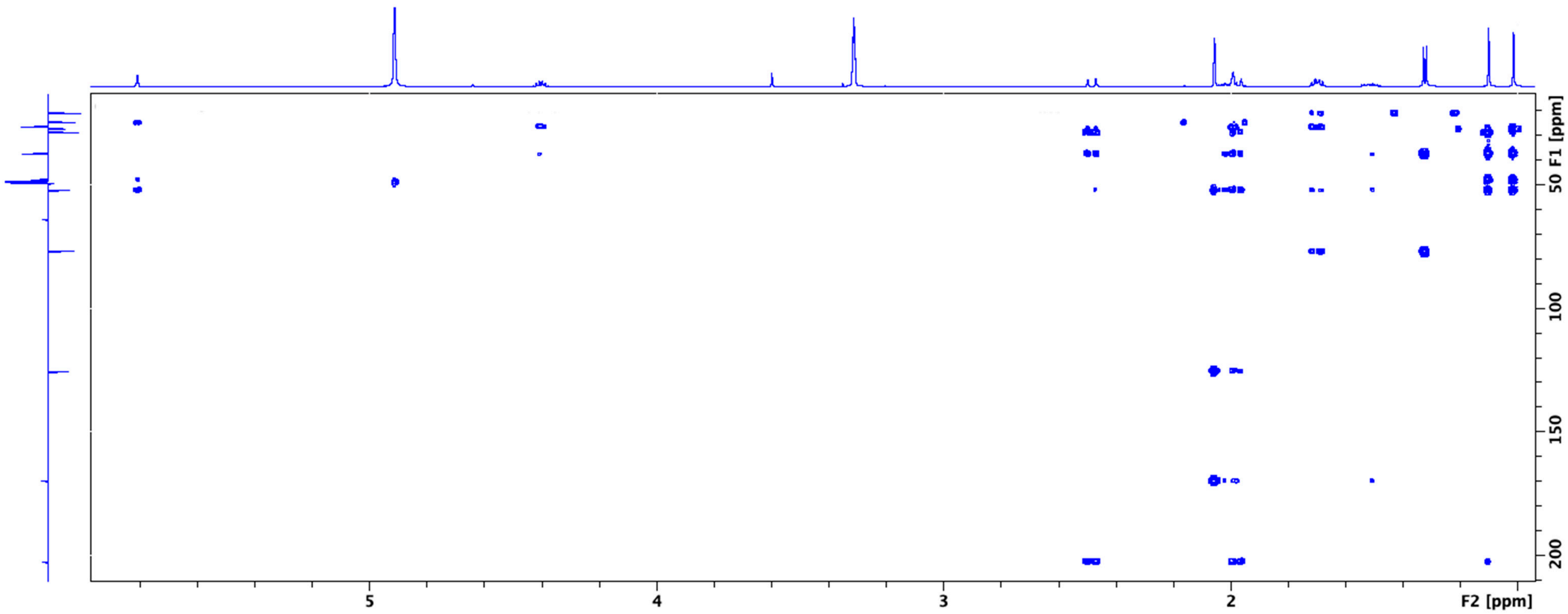

Figure S6. HMBC of **1** in CD_3OD.

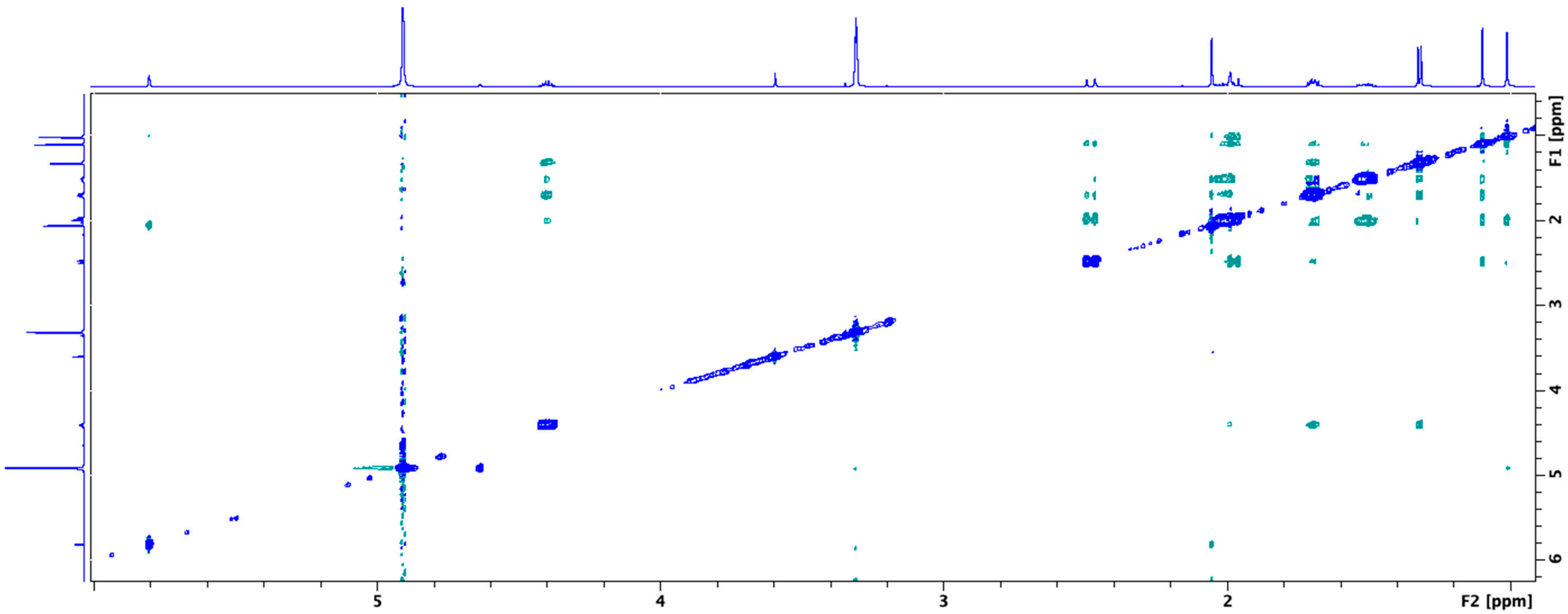

Figure S7. NOESY of **1** in CD_3OD.

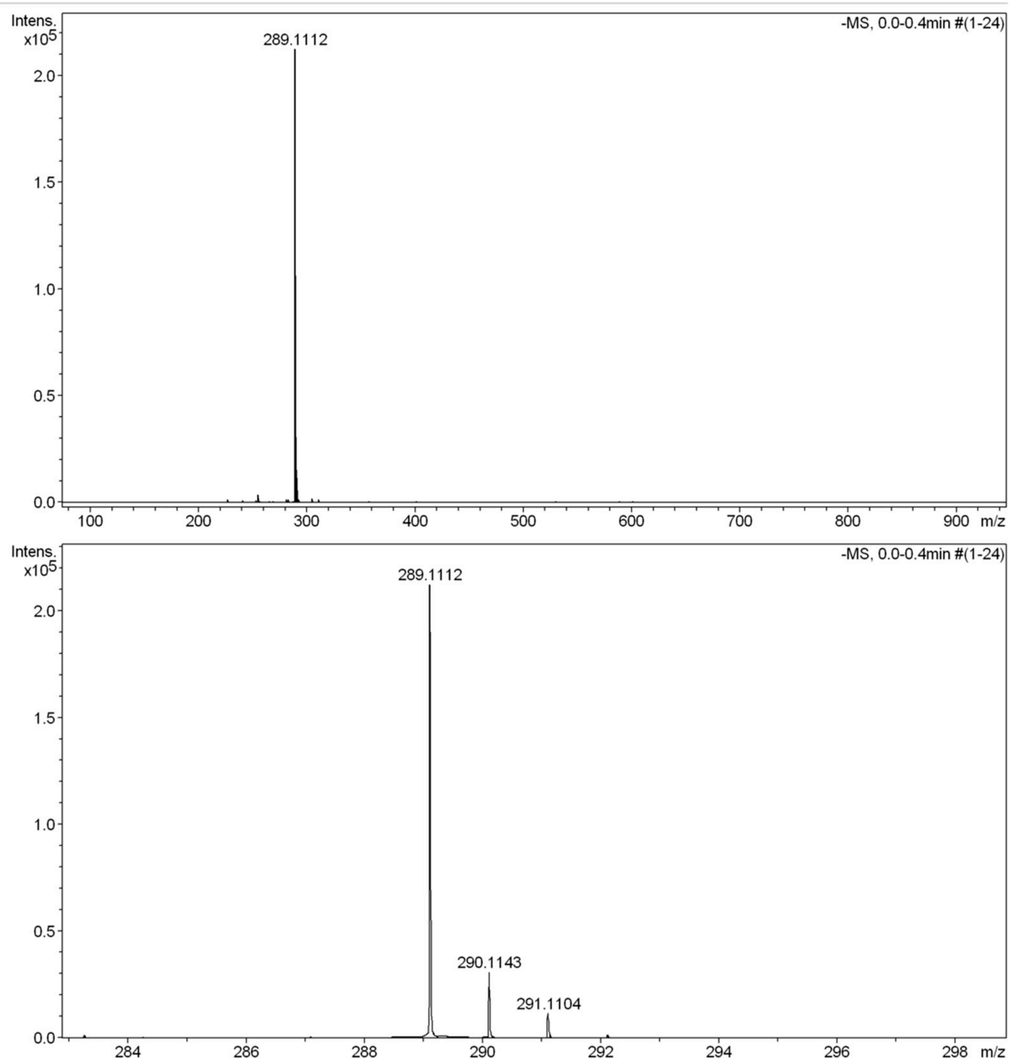

Figure S8. HR-ESI-TOF-MS (negative mode) of **1**.

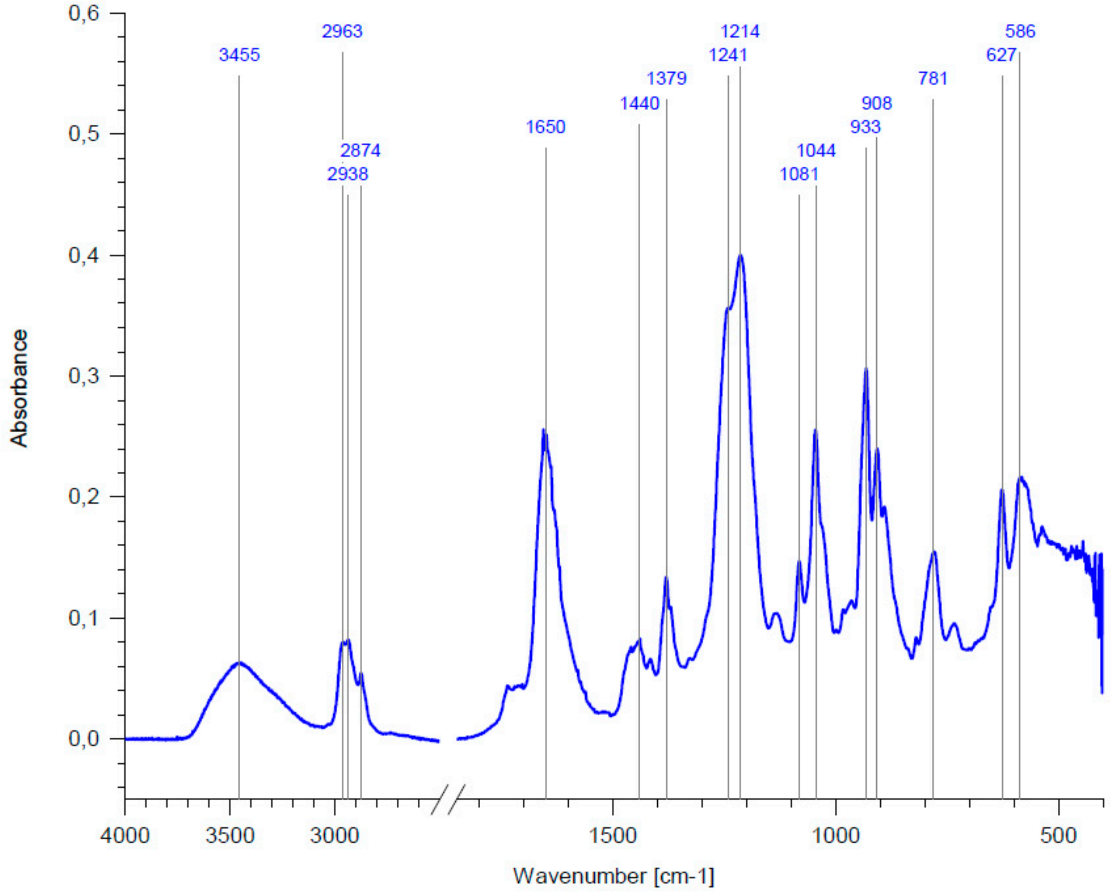

Figure S9. FT IR spectrum of **1**.

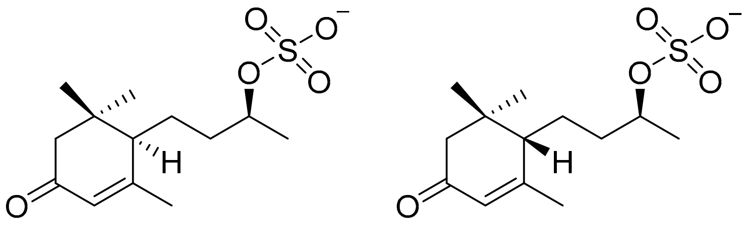

6*R**9*S**-**1a** 6*S**9*S**-**1b**

Figure S10. Two representative diastereomeric forms 6*R**9*S**-**1a** and 6*S**9*S**-**1b** used for conformation search based on molecular mechanics with MMFF force fields.

Table S1. DP4+ analysis results of 6*R**9*S**-**1a** (Isomer 1) and 6*S**9*S**-**1b** (Isomer 2).

Input	Isomer 1 (%)	Isomer 2 (%)
sDP4+ (H data)	44.69	55.31
sDP4+ (C data)	2.36	97.64
sDP4+ (all data)	1.92	98.08
uDP4+ (H data)	50.57	49.43
uDP4+ (C data)	50.37	49.63
uDP4+ (all data)	50.94	49.06
DP4+ (H data)	45.25	54.75
DP4+ (C data)	2.39	97.61
DP4+ (all data)	1.99	98.01

*Isomers 1 and 2 correlate to Figure S10

Table S2. Correlations between calculated (DP4+) and experimental ^{1}H and ^{13}C NMR chemical shifts of 6*R**9*S**-**1a** (Isomer 1) and 6*S**9*S**-**1b** (Isomer 2).

	^{13}C NMR chemical shifts (ppm)			^{1}H NMR chemical shifts (ppm)		
**No.**	**Exp.**	**Isomer 1**	**Isomer 2**	**Exp.**	**Isomer 1**	**Isomer 2**
1	26.7	29.1	28.0	1.99	2.52	2.58
2	29	30.0	28.8	2.48	2.76	2.94
3	37.7	35.6	35.3	1.97	2.26	2.45
4	77.1	85.6	88.2	5.81	6.42	6.20
5	21.2	23.1	21.8	1.10	1.40	1.38
6	48.1	49.0	48.6	1.01	1.28	1.28
7	202.4	198.1	197.3	2.05	2.41	2.49
8	125.4	119.3	123.0	1.51	2.16	1.94
9	169.9	174.6	171.5	2.02	2.11	2.14
10	52.3	50.8	52.0	1.69	1.96	1.92
11	37.3	42.8	40.7	4.40	4.72	4.67
12	27.5	29.7	27.7	1.32	1.68	1.68
13	24.9	27.8	28.3	-	-	-

*Isomers 1 and 2 correlate to Figure S10

Table S3. M06-2X/Def2SVP optimized lowest energy 3D conformers and energy analysis for compound **1**.

No.	3D conformers	G (Hartree)	ΔG (Kcal/mol)	Boltzmann distribution (%)
**1b-1**	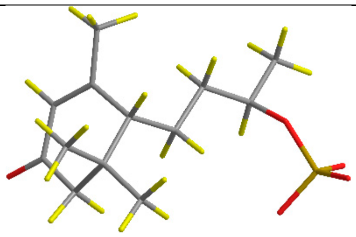	-1282.2608479	0.000000	44.58
**1b-2**	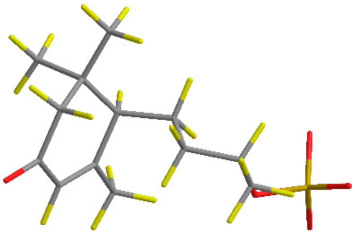	-1282.2608263	0.013554	43.57
**1b-3**	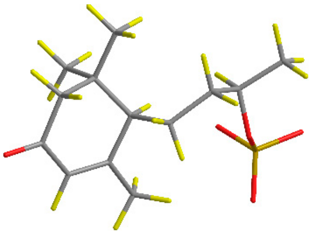	-1282.2588708	1.24063025	5.48
**1b-4**	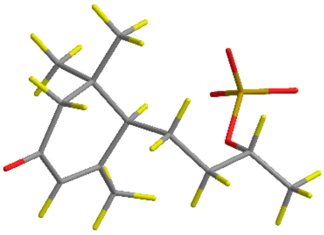	-1282.2588154	1.27539375	5.17
**1b-5**	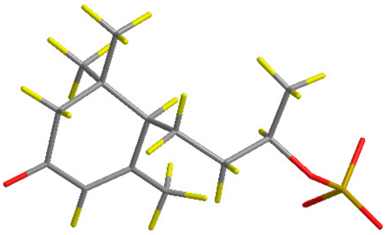	-1282.2574322	2.14335175	1.19

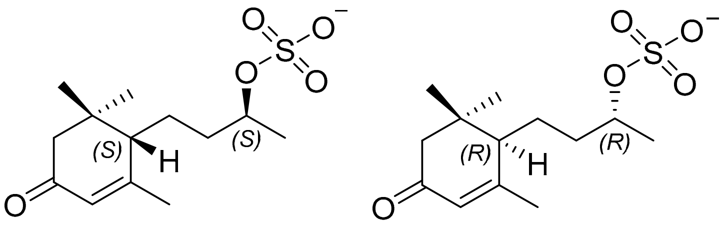

Figure S11. Two representative enatiomeric forms (6*S*,9*S*)-**1** and (6*R*,9*R*)-**1** used for conformation search based on molecular mechanics with MMFF force fields.

Blumenol C glucoside

Ground air-dried leaves of *Faramea tamberlikiana* subsp. *sessifolia* (130 g) were exhaustively extracted with methanol at room temperature (3 × 2 d) yielding 4 g crude extract. Partitioning was performed as described in the main text for isolation of compound **1** and yielded 1.5 g *n*-butanol phase. This extract was subjected to CC over silica gel 60 (40–60 µm particle size) eluted with mixtures of ethyl acetate and methanol. The fraction eluted with 20 % methanol (52.5 mg) was subjected to SEC and final purification by preparative TLC yielded 2.7 mg blumenol C glucoside.

White amorphous powder; MS, *m/z*: $[M+Na]^+$ 395.2038 (calcd for $[M+Na]^+$ 395.2040).

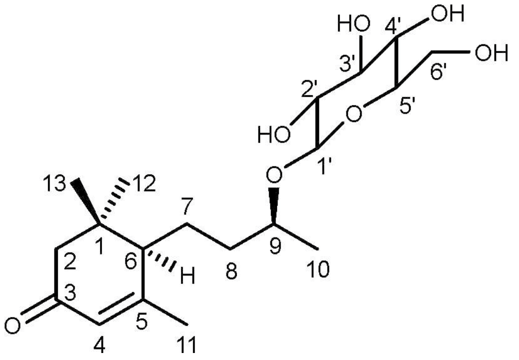

Figure S12. Structure of blumenol C glucoside.

Table S4. ^1H and ^{13}C NMR spectroscopic data for blumenol C glucoside in CD_3OD measured on a 600 MHz NMR. Numbering of carbon atoms is in accordance to Figure S12.

pos.	δ ^1H (ppm)	δ ^{13}C (ppm)
1	-	37.6, s
2	1.98 (d, 1H, 17.7), 2.48 (d, 1H, 17.7)	48.2, t
3	-	202.5, s
4	5.80 (m, 1H)	125.5, d
5	-	170.0, s
6	1.97 (m, 1H)	52.7, d
7	1.68 (m, 1H), 1.81 (m, 1H)	26.8, t
8	1.62 (m, 1H), 1.69 (m, 1H)	37.5, t
9	3.82 (m, 1H)	77.7, d
10	1.25 (d, 3H, 6.3)	22.0, q
11	2.04 (d, 3H, 1.3)	25.1, q
12	1.02 (s, 3H)	29.1, q
13	1.09 (s, 3H)	27.5, q
1’	4.31 (1H, d, 8.0)	104.1, d
2’	3.15 (1H, dd, 8.7, 8.0)	75.4, d
3’	3.33 (1H, dd, 8.7, 9.0)	78.4, d
4’	3.27 (1H, dd, 9.0, 8.4)	71.8, d
5’	3.25 (1H, m)	77.9, d
6’	3.65 (1H, dd, 12.3, 5.7)<br>3.85 (1H, dd, 12.2, 2.3)	62.9, t

Vomifoliol (= blumenol A)

Ground air-dried leaves of *Palicourea adusta* (124.5 g) were exhaustively extracted with methanol at room temperature (3 × 2 d) yielding 6.55 g crude extract which was partitioned as described in the main text for isolation of compound **1**. The chloroform, ethyl acetate and *n*-butanol phases were combined (650 mg) and subjected to repeated CC over silica gel 60 (0.2–0.5 mm particle size) with mixtures of petrol ether, ethyl acetate and methanol (46.1 mg) and 40–60 µm particle size with mixtures of chloroform and methanol. Final purification by preparative TLC afforded 2 mg vomifoliol also known as blumenol A.

White amorphous powder; MS, *m/z*: $[M+Na]^+$ 247.1302 (calcd for $[M+Na]^+$ 247.1304); Optical rotation: $[\alpha]_D^{20}$ (*c* 1.8 mg mL^{-1}, CH_3OH) = +93.9°.

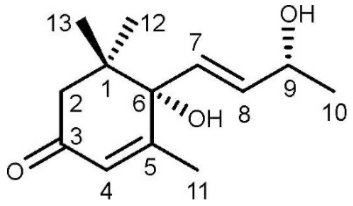

Figure S13. Structure of vomifoliol.

Table S5. ^1H and ^{13}C NMR of vomifoliol spectroscopic data in CD_3OD measured on a 600 MHz NMR. Numbering of carbon atoms is in accordance to Figure S13.

pos.	δ ^1H (ppm)	δ ^{13}C (ppm)
1	-	42.4, s
2	2.18 (d, 1H, 17.0), 2.50 (d, 1H, 17.0)	50.7, t
3	-	201.3, s
4	5.89 (m, 1H)	127.2, d
5	-	167.5, s
6	-	80.0, s
7	5.79 (d, 1H, 15.8),	130.0, d
8	5.82 (dd, 1H, 15.8, 5.1)	136.9, d
9	4.34 (dq, 1H, 5.1, 6.5)	68.7, d
10	1.26 (d, 3H, 6.5)	23.8, q
11	1.93 (d, 3H, 1.3)	19.6, q
12	1.06 (s, 3H)	23.4, q
13	1.03 (s, 3H)	24.5, q

Elemental analysis

Organic Elemental Micro-Analysis (EA) was originally developed by Fritz Pregl to characterize chemical substances and unveil their molecular composition [S1]. While the substance characterization is nowadays dominated by the subtle information gained by spectroscopic techniques characterization EA contributes complementary information to prove the purity of compounds. EA can also be applied to any material containing even a small fraction of organic substances. Service-requests related to carbon balance in environmental technology and biotechnology have become important applications of EA during the last decades.

The C/H/O-pattern of plant-derived material like the leaves of the investigated plants is generally dominated by the composition of cellulose ($C_6H_{10}O_5$ – 44.5 w-% C, 6.2 w-% H, 49.3 w-% O). The elements N and S are indicators of proteins and peptides as well as inorganic components (NH_4/NO_3, SO_4).

Figures S14–S17 show the data in a semi-logarithmic plot to resolve the characteristics of low level signals (base line) and the maximum level data (CO_2-peak) at the same time. There are four significant peaks assigned to nitrogen (N_2), carbon (CO_2), hydrogen (H_2O) and sulphur (SO_2). Numerical integration is done using linear base lines visible in the figures. A blank measurement is compensated by subtracting the trace prior to evaluation and plotting.

The diagrams include the evaluated raw data (area, A_X [V.s]), and the calibration factors f_X determined by a moving average model from calibration runs (third line). The blank file number is also indicated in the diagram. The analysis results w_X [w-%] and the sample weight m_0 [mg] are listed in the second line. Results are computed according to Equation S1.

$$w_X = \frac{f_X \cdot A_X}{m_0} \qquad \textit{Equ. S1}$$

In oxygen mode two peak areas are visible. The first sometimes splits into a part for hydrogen and nitrogen. Bothe cannot be used analytically. The third peak is assigned to oxygen (CO). The evaluation is done in the same way using Equation 1. Figures S14 and S15 show the raw data gained by analysis of AB 1 (elevated S-content) and AB 3 (normal S-content). Figures S16 and S17 represent examples from oxygen-determinations at the same two samples.

Ion analysis was done by capillary (free) zone electrophoresis using a CE 7100 system (Agilent). Separation was done at -30 kV along a 45 cm silica capillary with a 50 µm bore. The separation was performed at pH 9.1 in a CHES-Arg buffer (50 mM cyclohexylaminen-ethane sulfonic acid, 20 mM L-arginine) containing 1 mM tetradecyl-ammonium hydroxide as EOF modifier. The ion profile was recorded using a TraceDec conductivity detector. Evaluation was done using a series of mixed anion-standards made from 0.1 M stock solutions prepared from high purity chemicals and MilliQ-water. An amount of 1.5 to 2.5 mg of the grinded sample material were extracted with MilliQ-water and analyzed after filtration with 0.4 µm membrane filters.

Figure S18 shows the comparison of two samples with a 10 µM mixed anion standard containing bromide, chloride and sulfate. The analyte solutions contained about 2.6 mg of a sample in 50.0 mL water. Quantitative evaluation was based on peak areas. The calibration was based on a standard series from 5 to 100 µMol L^{-1}. Figure S19 shows the calibration for chloride and sulfate.

Table S6. Detailed microchemical elemental analysis data for sample A–E.

Sample	Anal-No	w-%C	w-%H	w-%N	w-%S	w-%O	w-%Cl	w-%SO4	H:C	C:N	C:S	N:S	C:O	C:Cl	S in SO_4	Sum	
A	210628/765	45,77	6,11	2,70	0,763				1,59	19,77	160,18	8,10					1,437 mg
	210628/766	45,77	6,11	2,69	0,744				1,59	19,85	164,27	8,28					1,308 mg
	210628/767	45,76	6,12	2,61	0,744				1,59	20,45	164,24	8,03					2,276 mg
	210714/148					41,72							1,46			97,02	
	210714/149					42,10							1,45			97,40	
	210714/150					40,93							1,49			96,23	
	210714/197					42,26							1,44			97,56	direct measured
	i210714/127						0,584	2,23							0,743		X93/739
	i210714/220						0,546	1,69							0,565		X93/744
	**Average**	**45,77**	**6,11**	**2,67**	**0,750**	**41,75**	**0,565**	**1,96**	**1,59**	**20,02**	**162,87**	**8,14**	**1,46**	**239,10**	**0,654**	**97,61**	
	σ	*0,01*	*0,01*	*0,05*	*0,011*	*0,59*	*0,027*	*0,38*									
	**theory**															**0,00**	
B	210628/768	47,11	6,25	2,06	0,503				1,58	26,68	250,09	9,37					1,064 mg
	210628/769	48,38	6,47	2,15	0,371				1,59	26,25	348,22	13,27					1,826 mg
	210628/770	48,49	6,43	2,19	0,359				1,58	25,83	360,67	13,96					1,281 mg
	210714/151					39,63							1,61			96,55	
	210714/152					38,51							1,66			95,43	
	210714/153					39,56							1,62			96,48	
	210714/198					40,70							1,57			97,62	direct measured
	i210714/130						0,260	1,54							0,515		X93/740
	i210714/223						0,274	1,40							0,468		X93/745
	**Average**	**47,99**	**6,38**	**2,13**	**0,411**	**39,60**	**0,267**	**1,47**	**1,58**	**26,24**	**311,81**	**11,88**	**1,61**	**530,57**	**0,491**	**96,79**	
	σ	*0,77*	*0,12*	*0,07*	*0,080*	*0,89*	*0,010*	*0,10*									
	**theory**															**0,00**	
C	210628/771	46,18	6,19	3,48	0,535				1,60	15,48	230,49	14,89					1,162 mg
	210628/772	46,07	6,24	3,33	0,532				1,61	16,14	231,24	14,33					2,019 mg
	210628/773	45,95	6,19	3,32	0,537				1,61	16,15	228,49	14,15					1,403 mg
	210714/154					40,08							1,53			96,26	
	210714/155					39,62							1,55			95,80	
	210714/156					40,04							1,53			96,22	
	210714/199					41,63							1,47			97,81	direct measured
	i210714/133						0,116	0,28							0,092		X93/741
	i210714/226						0,171	0,48							0,159		X93/746
	**Average**	**46,07**	**6,21**	**3,38**	**0,535**	**40,34**	**0,144**	**0,38**	**1,61**	**15,91**	**230,07**	**14,46**	**1,52**	**947,56**	**0,125**	**96,67**	
	σ	*0,12*	*0,03*	*0,09*	*0,003*	*0,88*	*0,039*	*0,14*									
	**theory**															**0,00**	
D	210628/774	46,10	6,32	4,17	0,549				1,63	12,90	224,23	17,39					1,322 mg
	210628/775	46,24	6,34	4,13	0,545				1,63	13,06	226,56	17,35					1,676 mg
	210628/776	48,88	6,74	4,14	0,570				1,64	13,77	228,99	16,63					1,792 mg
	210714/157					38,61							1,62			96,85	
	210714/158					38,00							1,65			96,24	
	210714/159					38,90							1,61			97,14	
	210714/200					40,19							1,56			98,43	direct measured
	i210714/136						0,425	< 0,05									X93/742
	i210714/229						0,332	< 0,05									X93/747
	**Average**	**47,07**	**6,47**	**4,15**	**0,555**	**38,93**	**0,379**	**< 0,05**	**1,64**	**13,24**	**226,62**	**17,11**	**1,61**	**367,10**		**97,54**	
	σ	*1,57*	*0,24*	*0,02*	*0,013*	*0,92*	*0,066*										
	**theory**															**0,00**	
E	210628/777	47,02	6,46	3,33	0,385				1,64	16,47	326,12	19,80					1,461 mg
	210628/778	47,20	6,41	3,37	0,386				1,62	16,34	326,52	19,98					1,326 mg
	210628/779	47,45	6,52	3,38	0,392				1,64	16,38	323,23	19,74					1,660 mg
	210714/160					39,66							1,59			97,09	
	210714/161					39,38							1,60			96,81	
	210714/162					39,29							1,60			96,72	
	i210714/139						0,321	< 0,05									X93/743
	i210714/232						0,306	< 0,05									X93/748
	**Average**	**47,22**	**6,46**	**3,36**	**0,388**	**39,44**	**0,314**	**< 0,05**	**1,63**	**16,40**	**325,28**	**19,84**	**1,59**	**444,62**		**97,19**	
	σ	*0,22*	*0,06*	*0,03*	*0,004*	*0,19*	*0,011*										
	**theory**															**0,00**	

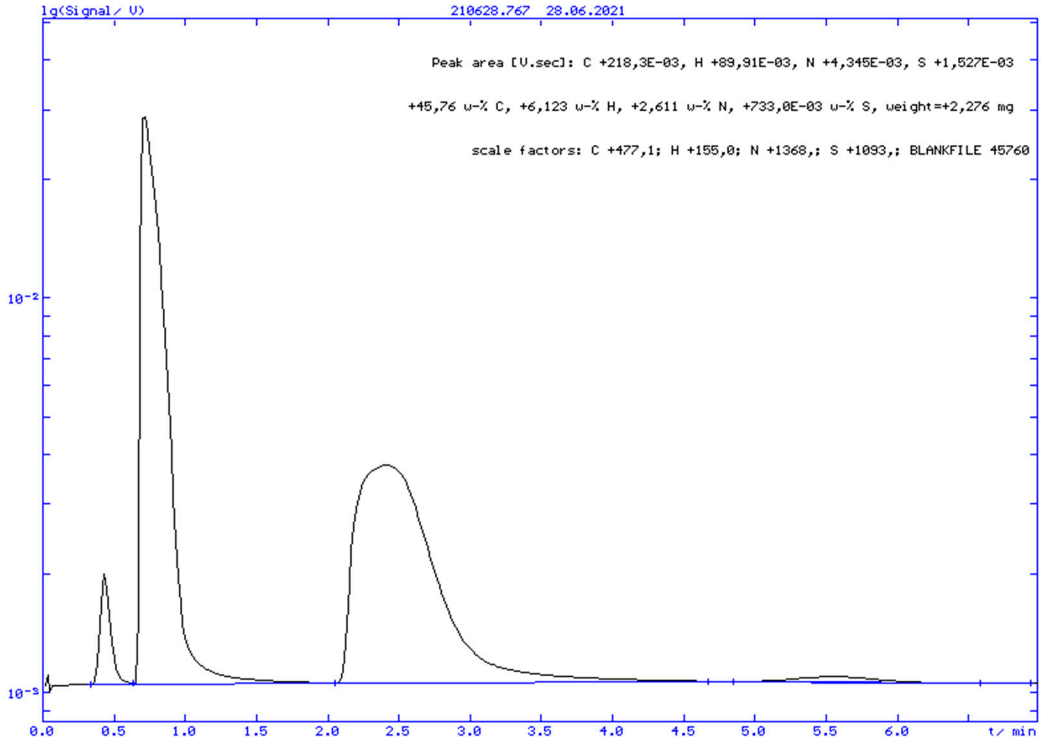

Figure S14. C/H/N/S-Analysis on EA 3000: TCD-trace of a C/H/N/S-run on sample A.

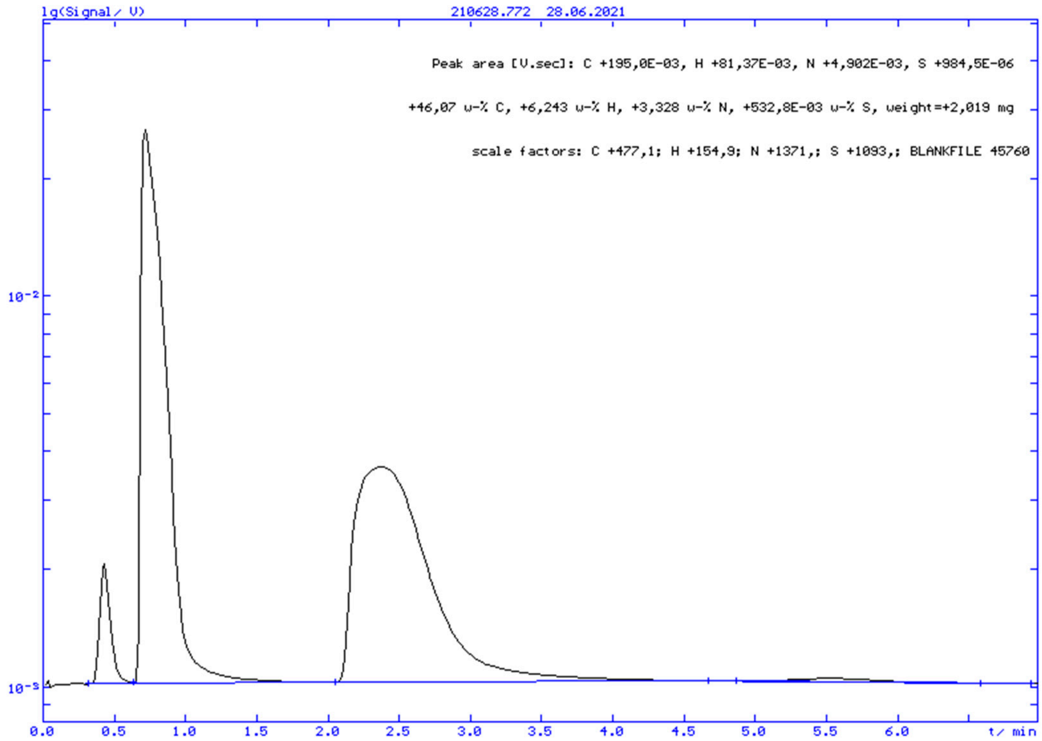

Figure S15. C/H/N/S-Analysis on EA3000: TCD-trace of a C/H/N/S-run on sample C.

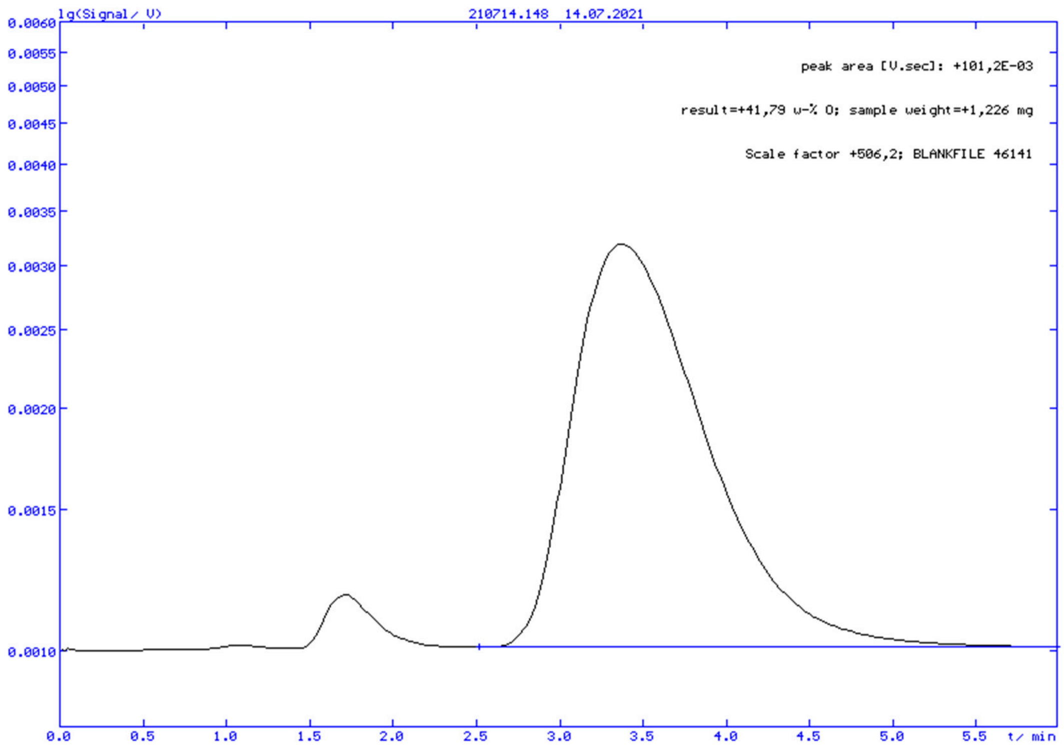

Figure S16. O-Analysis on EA3000 combined to the HT 1500 pyrolysis-system: TCD-trace of an analysis-run on sample A.

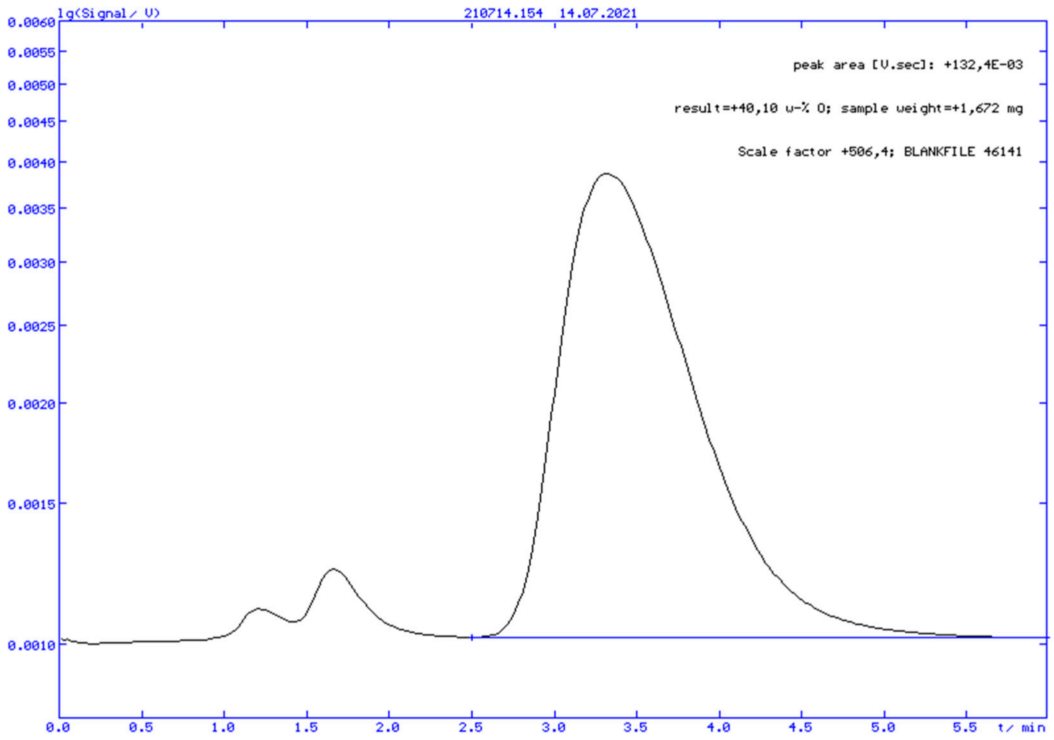

Figure S17. O-Analysis on EA3000 combined to the HT 1500 pyrolysis-system: TCD-trace of an analysis-run on sample C.

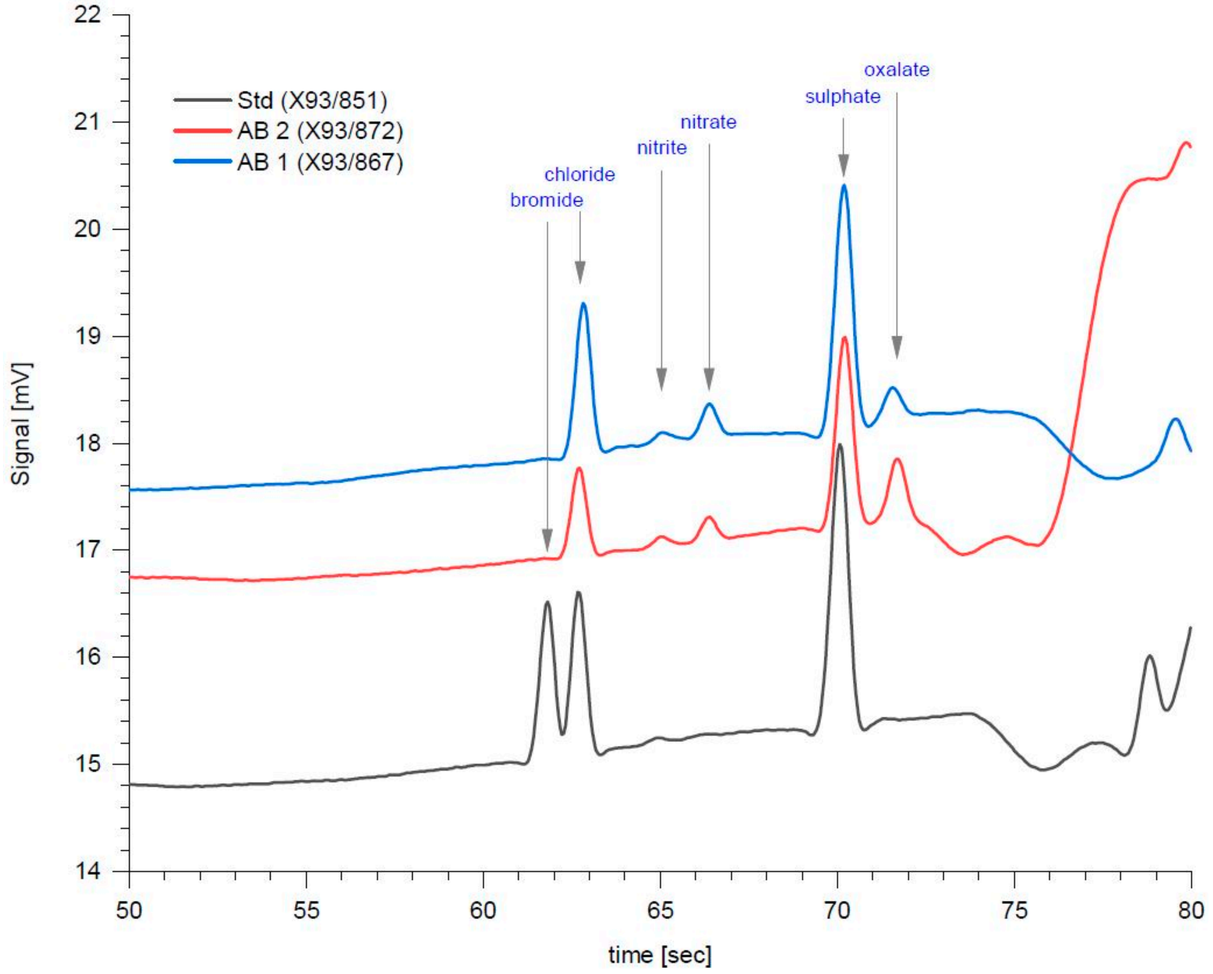

Figure S18. Comparison of two samples [A ≡ AB1; B ≡ AB2] with a 10 µM mixed anion standard containing bromide, chloride and sulfate.

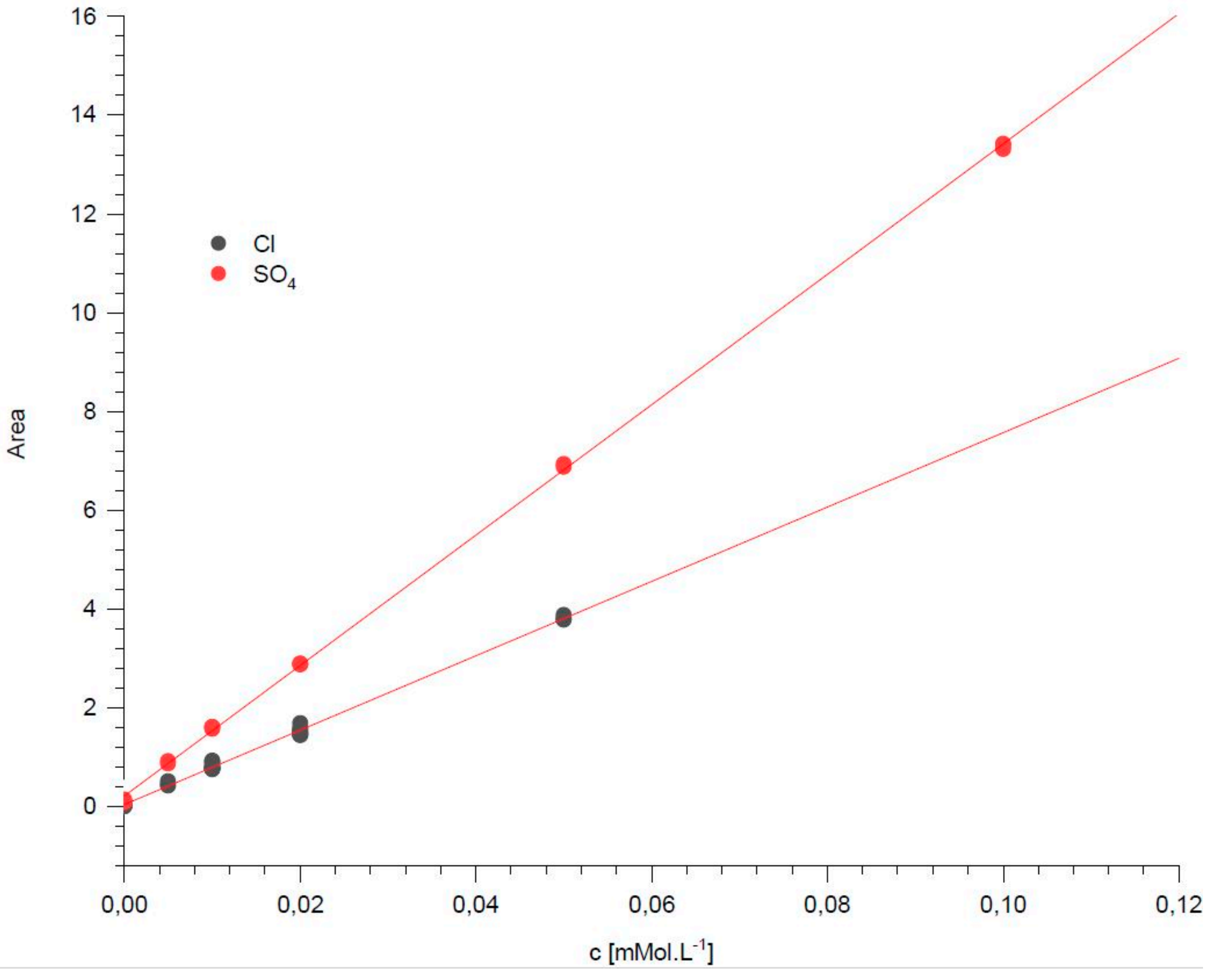

Figure S19. Calibration for chloride and sulfate.

References

S1. Pregl, F. *Die Quantitative Organische Mikroanalyse*; Julius Springer-Verlag: Berlin, 1917.
